# Acute:chronic workload ratio and load management for team sports: a multilevel meta-analysis

**DOI:** 10.3389/fpubh.2026.1896651

**Published:** 2026-08-13

**Authors:** Liangbi Ding, Anthony Weldon, Jiaqing Xu, Shane Malone, Jaime Sampaio, Zhiyong Zhang, Zhihui Zhou, Xiaoyi Liu, Shilong Han, Kai Xu, Yuanxing Wang, Peng Yuan

**Affiliations:** 1 School of Athletic Performance, Shanghai University of Sport, Shanghai, China; 2 Centre for Life and Sport Sciences, Birmingham City University, Birmingham, United Kingdom; 3 Aston Villa Foundation, Aston Villa Football Club, Birmingham, United Kingdom; 4 Department of Respiratory Science, University of Leicester, Leicester, United Kingdom; 5 Gaelic Sports Research Centre, School of Biological Health and Sport Science, Technological University Dublin-Tallaght Campus, Dublin, Ireland; 6 Research Center in Sports Science, Health Sciences and Human Development, CIDESD, Elite Research Community, Vila Real, Portugal; 7 Department of Sports Science, Exercise and Health, School of Life Sciences and Environment, University of Trás-os-Montes and Alto Douro, Vila Real, Portugal; 8 Police Sports and Tactic Training Department, Zhengzhou Police University, Zhengzhou, China

**Keywords:** acute:chronic workload ratio, injury prevention, injury risk, load monitoring, team sports

## Abstract

**Objective:**

The acute:chronic workload ratio (ACWR) has been widely used to monitor training load and injury risk in team-sport athletes. However, its practical utility remains debated because of methodological heterogeneity across load metrics, calculation methods, time-window definitions, sport types, and injury definitions. This systematic review and multilevel meta-analysis aimed to quantify the association between ACWR and injury risk in team-sport athletes and to examine potential moderators.

**Methods:**

Four electronic databases were searched from inception through October 2024. Prospective cohort studies or trials were eligible if they included team-sport athletes, reported associations between ACWR and injury outcomes, and provided sufficient data for effect size extraction or conversion. Study characteristics, participant information, injury definition, load metric, ACWR calculation method, time window, sport type, and effect estimates were extracted. Effect sizes were converted to Hedges’ g and pooled using a three-level random-effects model.

**Results:**

41 studies met the inclusion criteria, and 16 studies involving 797 athletes were included in the meta-analysis. Elevated ACWR showed a small-to-moderate association with higher injury risk (g = 0.35; 95% CI, 0.16–0.54), with substantial heterogeneity (I^2^ = 95.8%). Stronger associations were observed for contact and non-contact injuries, whereas effects were not significant for time-loss injuries or other moderators.

**Conclusion:**

Elevated ACWR may be associated with increased injury risk in specific contexts, but current evidence does not support its use as a stand-alone causal or predictive model. ACWR should be interpreted as a contextual monitoring indicator within individualized, multi-marker load-management systems.

**Systematic review registration:**

The systematic review was registered in PROSPERO under the unique identifier CRD420245127618.

## Graphical abstract

**Figure:**
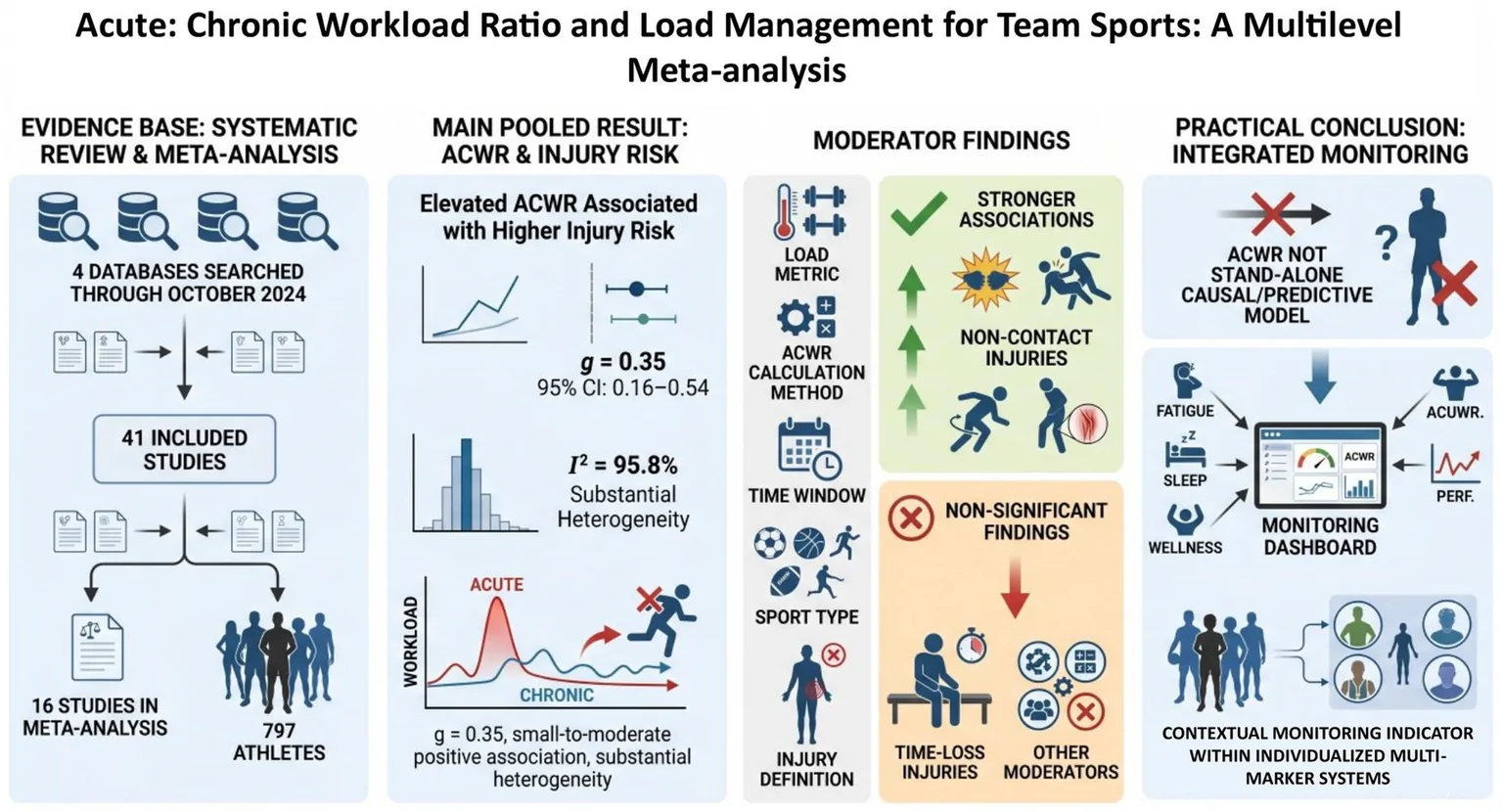

## Introduction

Training and competition loads in team sports such as soccer, rugby, and basketball fluctuate considerably over time. External load metrics describe the mechanical demands imposed on athletes, whereas internal load indicators reflect individual physiological responses to those demands (1, 2). Because the relationship between imposed workload and athlete response is nonlinear and influenced by contextual factors, inadequate monitoring may create a mismatch between training demands and athlete capacity, thereby compromising adaptation, preparedness, and injury resilience (1, 3, 4).

The Acute:Chronic Workload Ratio (ACWR) has been widely used in team sports to monitor relative changes in workload and to support load-management decisions (3, 5). Conceptually, ACWR compares recent workload with an athlete’s longer-term workload history and is often interpreted as an indicator of whether current training exposure is aligned with previous preparation (6, 7). However, ACWR is not a uniform construct. Rolling average (RA) and exponentially weighted moving average (EWMA) models, as well as coupled and uncoupled calculation approaches, differ in their assumptions and sensitivity to workload fluctuations (3, 5, 8).

Despite its widespread use, evidence regarding the association between ACWR and injury risk remains inconsistent. Early studies suggested that moderate ratios may be associated with lower injury risk, whereas excessive ratios may indicate elevated risk (5, 9). However, subsequent studies have reported mixed findings, ranging from limited predictive value to significant associations depending on the load metric, calculation method, and population examined (10, 11). These discrepancies suggest that the ACWR–injury relationship may not represent a universally stable effect, but may instead depend on methodological choices, sport-specific demands, and injury definitions. Several sources of heterogeneity may contribute to this inconsistency. Calculation method may influence sensitivity to short-term workload changes, while different team sports impose distinct mechanical and physiological demands that may alter the relevance of specific load metrics (12–14). In addition, time-window selection, coupling structure, player role, season phase, and injury classification may affect observed associations and limit the generalizability of current evidence (15–17). These issues are particularly important because multiple effect sizes are often derived from the same cohort, creating statistical dependence that may not be adequately addressed in conventional meta-analytic approaches.

Given the inconsistent evidence and the continued practical use of ACWR, a multilevel meta-analysis is needed to clarify its association with injury risk across methodological and sport-specific contexts. Therefore, this study aimed to estimate the overall association between ACWR and injury risk in team-sport athletes and to examine whether this relationship was moderated by sport type, injury classification, time window, load metric, and calculation approach. Rather than establishing ACWR as a definitive injury prediction tool, this study sought to define its methodological boundaries and clarify its potential value as a contextual indicator for training load management and athlete monitoring.

## Methods

### Design and search strategy

This systematic review was registered with PROSPERO (ID: CRD420245127618; registered 18 August 2025) and reported in accordance with the PRISMA 2020 statement (18). The search strategy was developed using the PICOS framework (19) (Supplementary Table S1). PubMed, Scopus, Web of Science, and SPORTDiscus via the EBSCOhost platform were searched from inception to 28 October 2024. Supplementary searches were conducted in Google Scholar, ResearchGate, and the reference lists of eligible studies. Search terms combined concepts related to ACWR, training load, injury risk, load management, and team sports using Boolean operators. Full search strategies are provided in Supplementary Table S5. Only English-language publications were included. Supplementary searches were conducted in Google Scholar, ResearchGate, and the reference lists of eligible studies; however, no dedicated search of grey-literature sources, such as theses, dissertations, conference proceedings, preprints, or institutional reports, was conducted. Only English-language publications were included.

### Study selection and eligibility criteria

**Figure 1:**
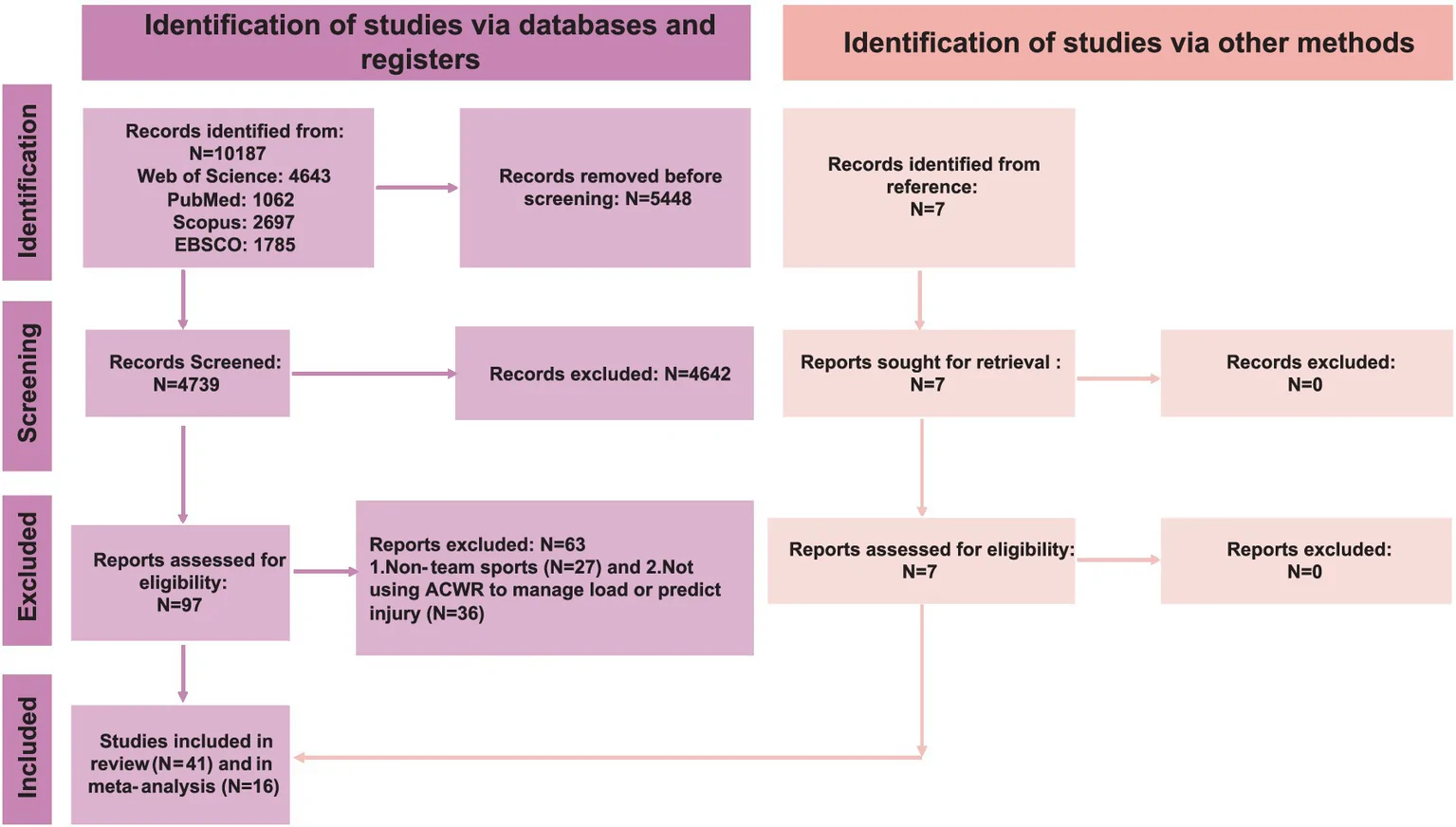
PRISMA flow diagram for study selection.

Two reviewers independently screened titles, abstracts, and full texts. Disagreements were resolved through discussion or third-reviewer adjudication. Eligible studies included competitive team-sport athletes, including professional, semi-professional, national-level, university-level, or elite youth athletes. Athlete competition level was classified according to previously published participant classification guidance, and elite youth athletes were considered eligible when they were involved in structured high-performance pathways or equivalent competitive contexts (20, 21). Studies were required to examine the association between ACWR and injury incidence or risk, report or allow derivation of ACWR values, and provide sufficient data for effect size extraction or conversion. Eligible designs included prospective or retrospective cohort studies, randomized controlled trials, and other longitudinal observational studies. Studies were excluded if they involved individual sports or recreational participants, examined workload ratios other than ACWR, lacked sufficient effect-size data, or were narrative reviews, conference abstracts, editorials, or case reports. The study selection process is shown in Figure 1.

### Data extraction and quality assessment

Data were extracted using a standardized form. Extracted information included study characteristics, participant characteristics, sport type, injury definition, ACWR calculation method, time window, load metric, and effect estimates. When multiple eligible effect sizes were reported within the same study, all were retained and modelled as nested observations in the multilevel meta-analysis to account for statistical dependence among effect sizes (22, 23). Potentially overlapping cohorts were assessed by comparing sample characteristics, study periods, and monitoring protocols; when substantial overlap was identified, the report with more complete data was retained. Predefined moderators included load metric type, ACWR calculation method, sport type, time window, and injury type.

Methodological quality was assessed independently by two reviewers. Cohort studies were evaluated using the Newcastle-Ottawa Scale, and randomized controlled trials were assessed using the Cochrane Risk of Bias 2 tool (24, 25). Disagreements were resolved through discussion or third-reviewer adjudication.

### Statistical analysis

Because the included studies reported different injury effect measures, effect sizes were converted to Hedges’ g with corresponding standard errors using established log-OR-to-SMD conversion procedures (26, 27). Original odds ratios and risk ratios are reported in the Supplementary materials to support practical interpretation. See Supplementary Table S7 for the study-level values. When standard errors, confidence intervals, or sample sizes were not directly available, missing values were derived using standard formulas.

A three-level random-effects meta-analysis was used to account for dependence among multiple effect sizes nested within studies (22, 23). Level 1 represented sampling variance, Level 2 represented within-study variance, and Level 3 represented between-study variance. Random intercepts were specified at Levels 2 and 3, allowing all eligible effect sizes to be retained without averaging. Heterogeneity was quantified using multilevel I^2^ statistics and τ^2^ variance components (28). Analyses were conducted in R using the metafor package, with variance components estimated by restricted maximum likelihood (29).

Subgroup analyses and meta-regression were conducted within the multilevel framework to examine predefined moderators. Subgroup analyses were performed when each category contained at least five effect sizes, and meta-regression was conducted when sufficient effect sizes were available, following common recommendations for moderator analyses in meta-analysis (28, 30). ACWR calculation method was retained as an exploratory moderator because of the limited number of eligible effect sizes and was interpreted cautiously. Between-group differences were tested using omnibus Q-tests, and regression coefficients with 95% confidence intervals were reported where applicable.

Publication bias was assessed using Egger’s linear regression test, a contour-enhanced funnel plot, and the trim-and-fill method (31, 32). Sensitivity analysis was performed using a leave-one-out procedure, in which each study was sequentially removed and the pooled effect size recalculated to assess robustness and identify influential studies (29).

## Results

### Literature search results

The systematic search yielded 10,187 records, with 5,448 remaining after duplicates were removed. After full-text screening, 41 studies were included in the qualitative synthesis and 16 in the meta-analysis. Among the 41 studies included in the qualitative synthesis, 25 were excluded from the meta-analysis because of insufficient effect-size data (k = 13), incompatible outcome measures (k = 8), or unavailable variance estimates (k = 4). Detailed exclusion rationales are provided in Supplementary Table S6. The study selection process is shown in Figure 1.

### Study characteristics and ACWR calculation features

Across the included studies, ACWR was calculated using both rolling-average (RA) and exponentially weighted moving-average (EWMA) methods. RA methods were reported in 13 studies (5, 9, 33–42), whereas EWMA methods were reported in 14 studies (7, 8, 33, 37, 40–49). Both methods were compared within the same dataset in nine studies (7, 8, 33, 37, 40–42, 44, 47). Detailed characteristics of all included studies are provided in Supplementary Table S9.

The included studies also differed in the use of coupled and uncoupled time-frame structures. Coupled models were reported in three studies (7, 8, 44), whereas uncoupled models were reported in five studies (7, 8, 44, 47, 50). Two studies directly compared coupled and uncoupled structures within the same dataset (7, 8). Because coupling structure was not uniformly reported and could overlap with time-window configuration, only computational method was included in formal moderator analysis.

Time-window configurations varied across studies. The 1:4-week ratio was most frequently adopted (*N* = 28) (5–9, 15, 34–36, 38–40, 42, 46–49, 51–57), followed by 1:3 weeks (*N* = 9) (8, 15, 36, 42, 45, 49, 52, 54, 55), and 1:2 weeks (*N* = 6) (8, 15, 36, 42, 45, 52).

Training load metrics were categorized as internal, external, or combined. Internal metrics, typically based on the session rating of perceived exertion (sRPE), were used in 26 studies (9, 15, 33, 35–40, 41, 44, 46–50, 52–55, 57–61). External metrics, often derived from global positioning system (GPS), were used in 21 studies (4, 5, 8, 34, 35, 37–39, 40, 43–45, 51, 55, 58, 60, 62–64). Eight studies used both internal and external metrics (35, 37–39, 44, 55, 60, 65).

### Primary analyses

**Figure 2:**
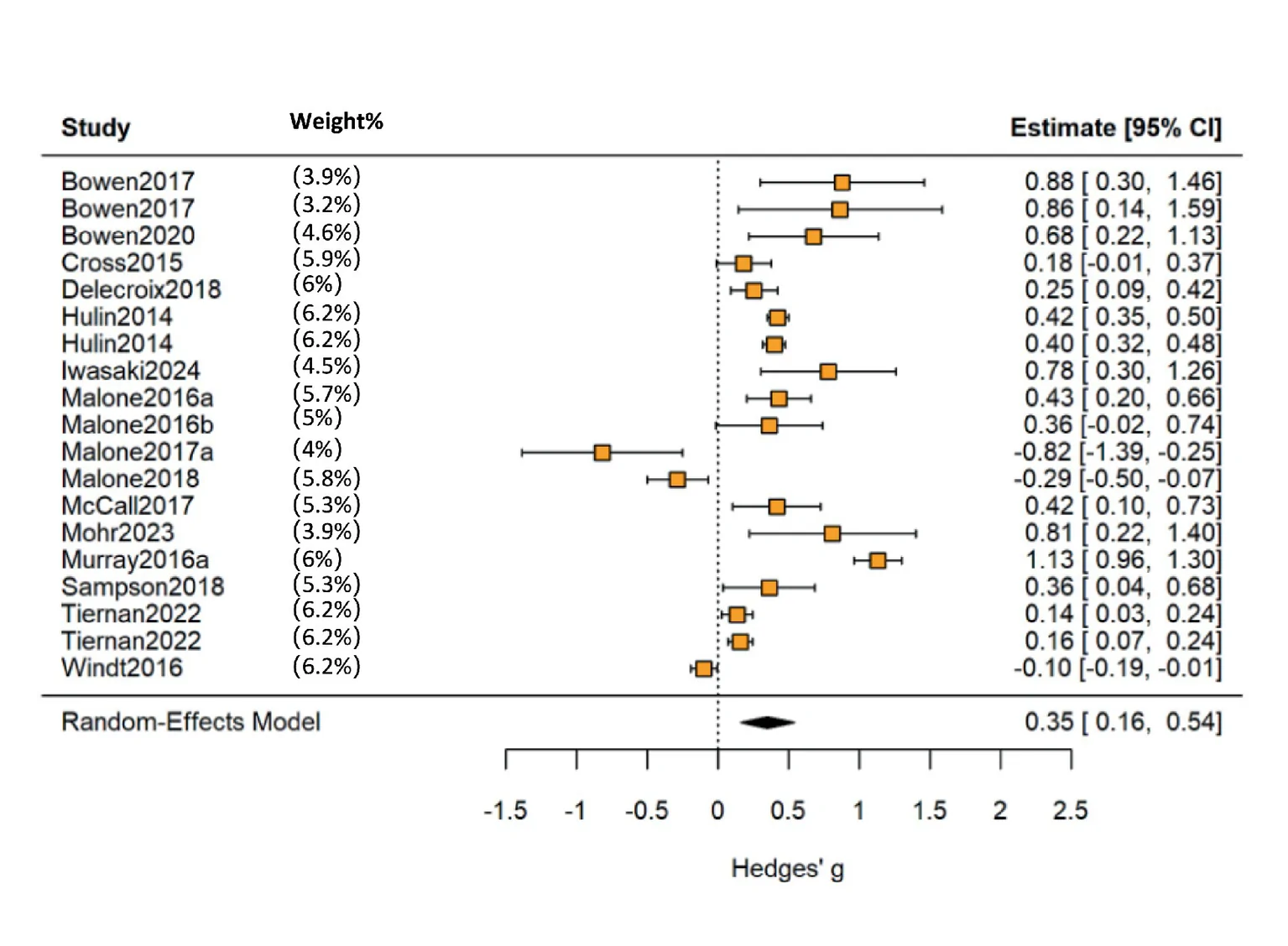
Forest plot of the association between acute:chronic workload ratio (ACWR) and injury risk.

The three-level meta-analysis showed a significant positive association between elevated ACWR and injury risk (Hedges’ g = 0.35, 95% CI 0.16 to 0.54, *p* < 0.001), indicating a small-to-moderate effect. Substantial heterogeneity was observed (I^2^ = 95.8%). Variance decomposition showed that 42% of heterogeneity was attributable to within-study differences (τ^2^ = 0.073) and 58% to between-study variance (τ^2^ = 0.101), indicating that the magnitude of the ACWR–injury association varied considerably across studies and analytic contexts. The pooled analysis is shown in Figure 2.

### Moderators and meta-regression

**Figure 3:**
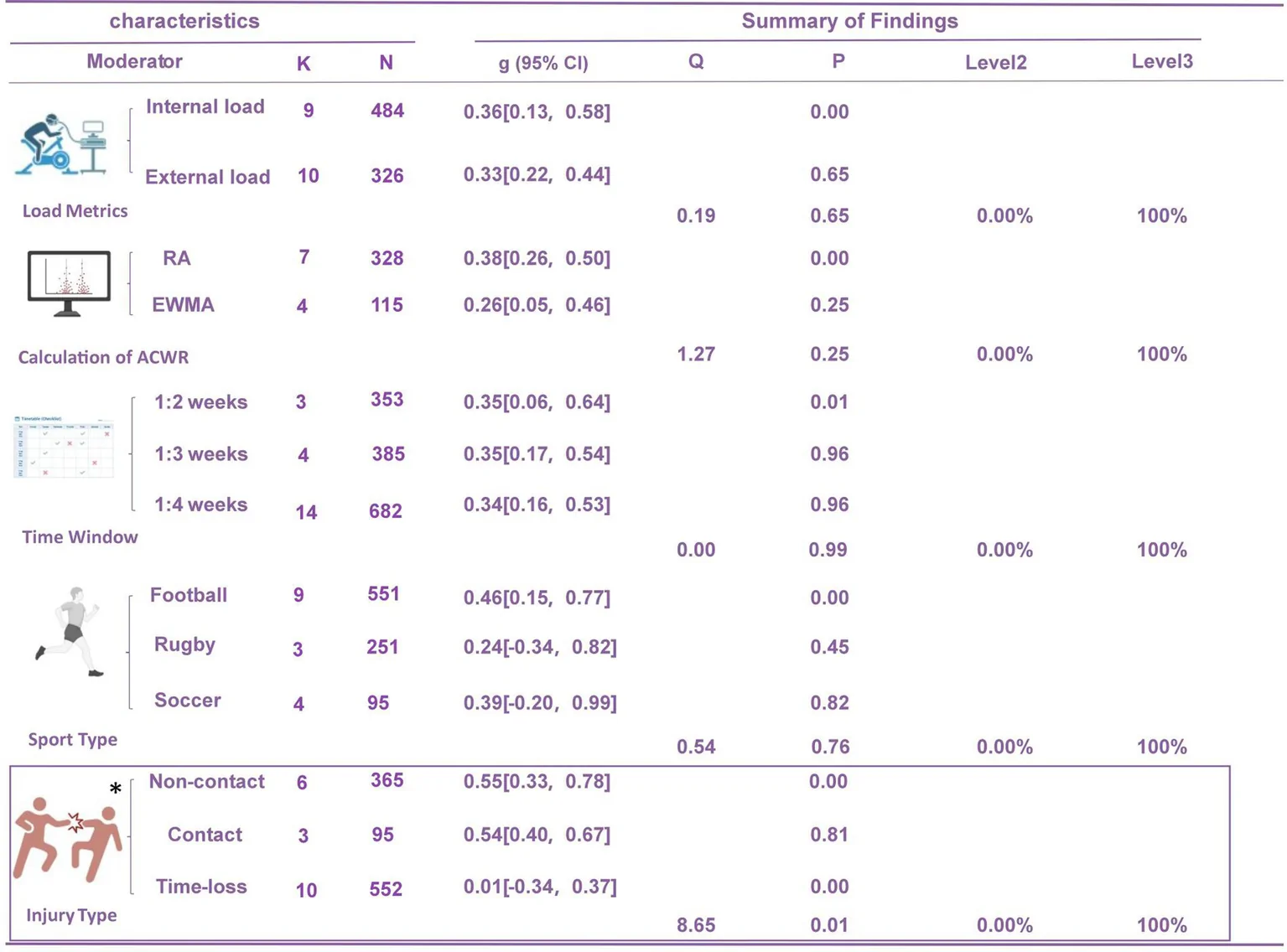
Summary of subgroup analyses examining the moderating effects of load type, calculation method, time window, sport type, and injury type on the relationship between ACWR and injury risk. Asterisk and shaded box highlight the only statistically significant moderator (injury type).

Five moderators were examined. Injury type was the only significant moderator of the ACWR–injury association, whereas load type, calculation method, time window, and sport type were not significant moderators. A summary of the subgroup analyses is shown in Figure 3, with detailed moderator statistics and complementary visualizations provided in Supplementary Table S4 and Supplementary Figures S1, S2.

Load type was not a significant moderator (QM = 0.19, df = 1, *p* = 0.65), and pooled effects were similar for internal (g = 0.36, 95% CI 0.13 to 0.58; *N* = 484) and external load metrics (g = 0.33, 95% CI 0.22 to 0.44; *N* = 326). Calculation method was also not significant (QM = 1.27, df = 1, *p* = 0.25), although the pooled effect was numerically larger for RA (g = 0.38, 95% CI 0.26 to 0.50; *N* = 328) than for EWMA (g = 0.26, 95% CI 0.05 to 0.46; *N* = 115). Time window did not significantly moderate the association (QM = 0.00, df = 2, *p* = 0.99), with similar pooled effects for 1:2-week (g = 0.35, 95% CI 0.06 to 0.64; *N* = 353), 1:3-week (g = 0.35, 95% CI 0.17 to 0.54; *N* = 385), and 1:4-week configurations (g = 0.34, 95% CI 0.16 to 0.53; *N* = 682). Sport type was not significant (QM = 0.54, df = 2, *p* = 0.76), although football showed the largest numerical effect (g = 0.46, 95% CI 0.15 to 0.77; *N* = 551), followed by soccer (g = 0.39, 95% CI − 0.20 to 0.99; *N* = 95) and rugby (g = 0.24, 95% CI − 0.34 to 0.82; *N* = 251).

Injury type significantly moderated the ACWR–injury association (QM = 8.65, df = 2, *p* = 0.01), accounting for 35.95% of between-study heterogeneity. Pooled effects were similar for contact injuries (g = 0.54, 95% CI 0.40 to 0.67) and non-contact injuries (g = 0.55, 95% CI 0.33 to 0.78), whereas time-loss injury outcomes showed a near-null effect (g = 0.01, 95% CI −0.34 to 0.37).

### Methodological quality, sensitivity analysis, and publication bias

**Figure 4:**
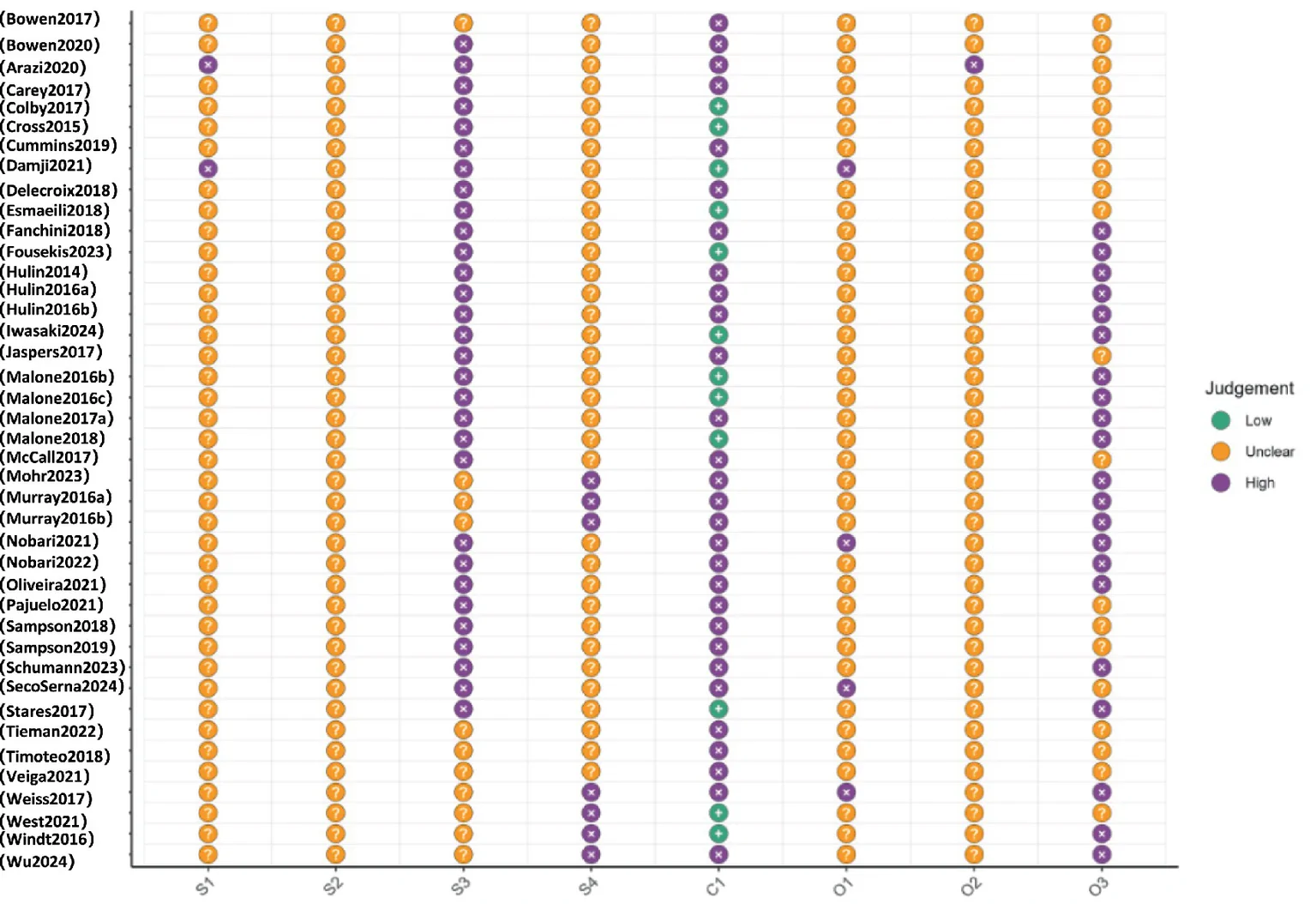
Risk of bias summary across all included studies, assessed using a modified Newcastle–Ottawa scale. The evaluation covered three domains: selection (S1–S4), comparability (C1), and outcome (O1–O3). Specifically, S1 assessed cohort representativeness, S2 evaluated the accuracy of exposure measurement, S3 examined whether outcomes were absent at baseline, and S4 addressed the clarity of outcome definitions. C1 assessed whether key confounding factors were controlled. Outcome-related domains included O1 (assessment method), O2 (adequacy of follow-up duration), and O3 (completeness of follow-up). Risk of bias was rated as low (green), unclear (orange), or high (purple).

Among the 41 cohort studies assessed using the Newcastle–Ottawa Scale, 80% (k = 33) were rated as moderate quality, 10% (k = 4) as high quality, and 10% (k = 4) as low quality (Supplementary Table S10). The risk-of-bias summary showed that the most common methodological limitations involved inadequate control of confounding factors, short or insufficiently reported follow-up duration, and limited reporting of cohort representativeness, recruitment procedures, or outcome assessment (Figure 4).

**Figure 5:**
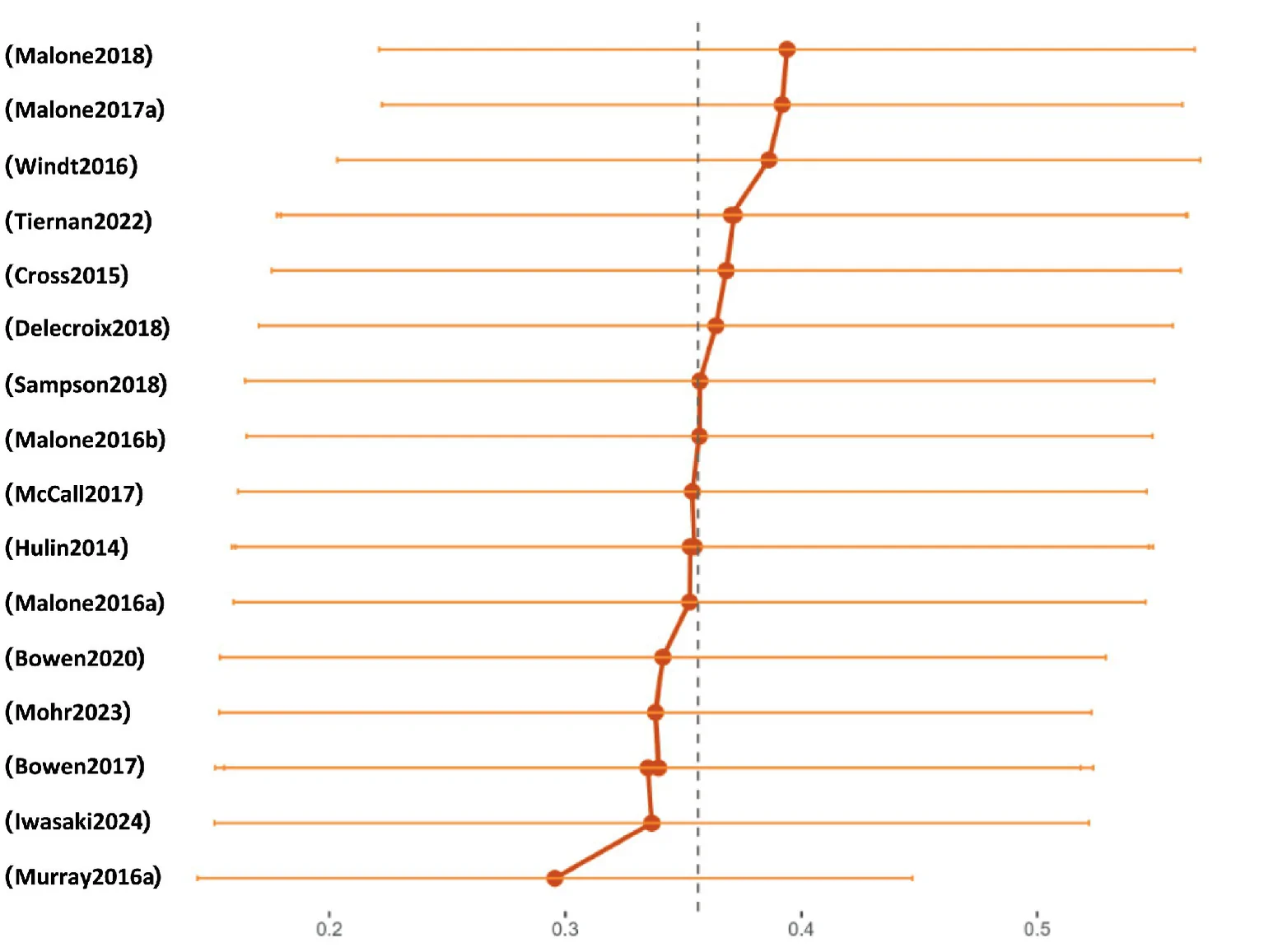
Leave-one-out sensitivity analysis of the association between acute:chronic workload ratio (ACWR) and injury risk.

**Figure 6:**
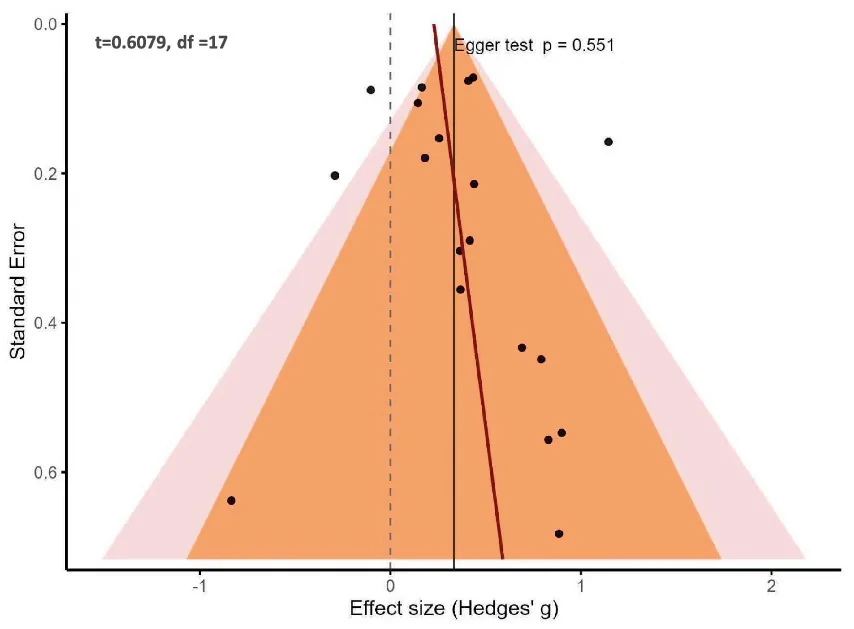
Funnel plot for publication bias assessment of the association between acute:chronic workload ratio (ACWR) and injury risk.

Leave-one-out sensitivity analyses showed that the pooled effect size remained stable across iterations, ranging from 0.33 to 0.37, indicating that no single study had a disproportionate influence on the overall estimate (Figure 5). The funnel plot was approximately symmetrical (Figure 6). Egger’s test showed no evidence of small-study effects (t = 0.61, df = 17, *p* = 0.55), and the trim-and-fill procedure identified no missing studies.

## Discussion

The present multilevel meta-analysis found a significant but modest positive association between elevated ACWR and injury risk in team-sport athletes. However, substantial heterogeneity across studies indicates that ACWR should not be interpreted as a stand-alone injury prediction tool. Rather, the findings suggest that ACWR may reflect deviations from recent training progression and should be used as a contextual monitoring indicator within broader athlete-management systems. This interpretation is consistent with previous studies reporting associations between higher ACWR and injury risk, while also acknowledging the limitations of using a single workload ratio to explain complex and multifactorial injury outcomes (3, 6, 53).

This cautious interpretation is reinforced by the possibility of reverse causation, which remains a central challenge for causal inference in ACWR research. Although ACWR is generally treated as an exposure preceding injury, early symptoms, subclinical tissue impairment, or reduced readiness may prompt athletes or practitioners to modify training before an injury is formally diagnosed. Such pre-injury modification can alter the acute load and, depending on the calculation window, the chronic load, meaning that the resulting ratio may partly reflect an evolving injury process rather than an independent cause of injury. Previous studies attempted to reduce this bias by excluding workload data immediately before injury, but the exclusion windows varied, including 3 days in Hulin et al. and 7 days in Hulin et al. (5) and Malone et al. (9). This inconsistency leaves uncertainty regarding the temporal sequence between workload change and injury development. Consequently, the present observational evidence cannot establish that an elevated ACWR causes injury or that reducing the ratio would necessarily reduce injury risk. Future prospective studies should record early symptoms and the reasons for training modification, apply clearly defined pre-injury lag periods, and model workload as a time-varying exposure.

The moderator analyses further highlight the context-dependent nature of ACWR. Load type was not a significant moderator, suggesting that internal and external metrics showed broadly similar associations with injury risk. However, this should not be interpreted as evidence that these metrics are interchangeable. Internal measures such as session-RPE capture perceived physiological and psychological strain but may be affected by subjective and contextual factors (66, 67). External measures derived from GPS provide objective information on mechanical demands such as running distance, high-speed running, and acceleration profiles, but may not fully capture internal strain during technically demanding or cognitively demanding activities (68–71). Therefore, metric selection should be guided by the injury mechanism of interest, sport-specific demands, and the purpose of monitoring rather than by the assumption that one metric category is universally superior.

Similarly, calculation method and time-window configuration did not significantly moderate the ACWR–injury association. Although EWMA may theoretically be more sensitive to recent workload fluctuations than RA, this advantage did not translate into a significantly stronger association with injury risk in the present analysis (40). This may reflect limited subgroup power, differences in parameter specification, or the possibility that method selection has less influence than the quality and relevance of the load metric itself. Likewise, no clear difference was observed across 1:2, 1:3, and 1:4 week configurations. Shorter windows may be more responsive to rapid workload changes during return-to-play or fixture congestion, whereas longer windows may better reflect cumulative exposure but may be less sensitive to acute fluctuations (3, 12, 38, 42, 72). These findings suggest that ACWR calculation should be selected according to athlete status, training phase, data frequency, and monitoring purpose, rather than according to a universal model or threshold.

Injury type was the only significant moderator. Contact and non-contact injuries showed positive associations with ACWR, whereas time-loss injury outcomes showed a near-null effect. This finding suggests that broad injury definitions may obscure the relationship between workload change and specific injury mechanisms. Time-loss classifications often combine acute traumatic injuries, overuse injuries, and conditions with different causal pathways, which may dilute workload-related associations. From a practical perspective, ACWR may be most informative when injury outcomes are classified according to mechanism and load sensitivity. For example, non-contact and overuse-related injuries may be more directly linked to rapid workload changes, whereas contact injuries are also strongly influenced by match context, opponent behaviour, and collision exposure (5, 52).

Sport type was not a significant moderator, although numerical differences were observed across sports. Football showed the largest effect, whereas rugby showed a weaker association. This pattern may reflect differences in sport-specific injury mechanisms, with rugby involving a larger contribution from contact and collision exposure, while football and soccer may be more sensitive to high-intensity running and multidirectional workload changes (8, 51, 59, 62). Nevertheless, the limited number of studies within each sport subgroup reduces confidence in between-sport comparisons. Therefore, current evidence does not support sport-specific ACWR thresholds, but it does suggest that ACWR values should be interpreted in relation to sport demands, player position, training history, and contextual exposure.

Taken together, the moderator findings should be interpreted in the context of the very high overall heterogeneity (I^2^ = 95.8%). The absence of statistically significant differences for most predefined moderators does not imply that the ACWR–injury association is consistent across settings; rather, the examined variables explained only part of the observed variation. Residual heterogeneity may reflect differences in competition congestion, player position, sex, age, training history, physical capacity, return-to-play status, monitoring frequency, and season phase. These factors can influence both workload exposure and an athlete’s capacity to tolerate that exposure. For example, congested schedules may reduce recovery opportunities, position-specific demands may produce different mechanical and physiological loading patterns, and athletes returning from injury may have modified training programmes and unstable chronic-load histories. Similarly, the meaning of a short-term workload change may differ between preseason, congested in-season periods, and the end of the competitive season. Because these characteristics were not reported consistently across the included studies, their moderating effects could not be examined formally. The pooled estimate should therefore be viewed as an average across heterogeneous sporting and monitoring contexts rather than as an effect that can be generalized uniformly to all athletes.

## Limitations

Several limitations should be considered. First, although the three-level model allowed multiple effect sizes nested within studies to be retained, residual statistical dependence may have remained when multiple outcomes, time windows, or workload metrics were derived from the same cohort. Second, several moderator analyses were based on a limited number of studies and effect sizes, reducing statistical power to detect potentially meaningful subgroup differences; therefore, non-significant moderator results should not be interpreted as evidence of equivalence. Third, the very high residual heterogeneity indicates that the pooled estimate represents an average across diverse athlete populations, sports, monitoring protocols, and competitive contexts. Fourth, variability in injury definitions, ACWR calculation procedures, and pre-injury exclusion windows limited comparability across studies. Finally, because the included evidence was observational and reverse causation remains possible, causal conclusions cannot be drawn regarding whether changes in ACWR directly increase injury risk.

The evidence base was also restricted to English-language publications, and grey-literature sources were not systematically searched. Although supplementary searches were conducted in Google Scholar and ResearchGate and the reference lists of eligible studies were examined, relevant unpublished or non-indexed studies may have been missed. These restrictions may have introduced language or publication bias and reduced the comprehensiveness of the review. Although Egger’s test and the trim-and-fill procedure did not indicate small-study effects, these methods may have limited power in a small and highly heterogeneous evidence base and cannot account for studies that were never retrieved. Publication bias therefore cannot be excluded solely on the basis of the negative statistical tests.

### Future research

Future research should move beyond universal ACWR cut-points by developing individualized, dynamically updated reference ranges that account for training history, sport, player position, season phase, physical capacity, and return-to-play status. Such approaches require large prospective cohorts with repeated within-athlete measurements and should be validated across independent teams, seasons, and competitive levels before clinical use. Future studies should also standardize injury definitions, workload metrics, calculation procedures, and pre-injury lag periods, while recording early symptoms and the reasons for training modification so that workload can be modelled as a time-varying exposure.

Machine-learning and Bayesian approaches may help characterize nonlinear relationships, interactions among risk factors, and changes in an athlete’s risk profile as new longitudinal observations become available. However, improved model fit should not be assumed to indicate clinical usefulness. Predictive studies should use adequate numbers of injury events, prespecified analysis plans, transparent handling of missing data, and rigorous assessment of discrimination, calibration, and clinical utility. Models should undergo both internal and external validation, be compared with simpler approaches, and test whether adding ACWR provides incremental information beyond established predictors. Longitudinal monitoring systems should integrate internal and external workload with physiological, biomechanical, wellness, previous injury, competition schedule, and clinical data. This would clarify whether ACWR contributes unique decision-relevant information or primarily reflects signals already captured by other monitoring variables.

### Clinical and practical implications

The pooled effect size of Hedges’ g = 0.35 represents a small-to-moderate standardized association between ACWR and injury outcomes and should not be interpreted as a 35% increase in injury risk. Hedges’ g was used to synthesize effect estimates reported in different forms, including odds ratios and risk ratios, on a common standardized scale. However, this pooled estimate cannot be translated into a single absolute change in injury probability without a common baseline injury risk, exposure distribution, injury definition, and reference category. Because these characteristics varied across the included studies, the original odds ratios and risk ratios are retained in the Supplementary materials to support study-specific interpretation. At the team or squad level, an association of this magnitude may still be practically relevant in high-exposure environments, including elite sport, where athletes experience repeated training and competition demands and the cumulative injury burden may be substantial. Nevertheless, the pooled association does not by itself demonstrate sufficient individual-level discrimination for injury prediction or justify a universal intervention threshold.

Accordingly, ACWR is best used as a contextual flag within an individualized, multi-marker monitoring system rather than as a diagnostic or stand-alone decision-making tool. An unusual change in ACWR may prompt practitioners to review the athlete’s recent workload progression, symptoms, recovery and wellness status, previous injury, neuromuscular function, competition schedule, and sport-specific exposure before adjusting training. Conversely, ACWR should not be used in isolation to determine whether an athlete should train, compete, or return to play. Commonly cited thresholds such as 0.8–1.3 or >1.5 may serve as heuristic reference points, but their meaning depends on the athlete, sport, workload metric, injury definition, and monitoring context. The practical value of ACWR therefore lies in identifying workload deviations that warrant further assessment, while final decisions remain informed by complementary monitoring data and clinical judgment. A practical decision framework for interpreting and responding to ACWR is summarized in Supplementary Table S8.

## Conclusion

This systematic review and multilevel meta-analysis identified a significant but modest positive association between elevated ACWR and injury risk in team-sport athletes. However, substantial heterogeneity and limited moderator effects indicate that ACWR should not be used as a stand-alone injury prediction tool or interpreted through universal thresholds. Injury type was the only significant moderator, suggesting that mechanism-based injury classification is important when interpreting workload–injury relationships. In practice, ACWR may be best used as a contextual monitoring indicator within individualized, multi-marker load-management systems that consider athlete readiness, training history, sport-specific demands, and injury mechanisms. Future research should adopt standardized prospective designs and evaluate whether ACWR adds practical value beyond other monitoring indicators.

## Data Availability

The original contributions presented in the study are included in the article/Supplementary material, further inquiries can be directed to the corresponding author.

## References

[1] Vanrenterghem J Nedergaard NJ Robinson MA Drust B. Training load monitoring in team sports: a novel framework separating physiological and biomechanical load-adaptation pathways. Sports Med. (2017) 47:2135–42. doi: 10.1007/s40279-017-0714-2, 28283992

[2] McLaren SJ Macpherson TW Coutts AJ Hurst C Spears IR Weston M. The relationships between internal and external measures of training load and intensity in team sports: a meta-analysis. Sports Med. (2018) 48:641–58. doi: 10.1007/s40279-017-0830-z, 29288436

[3] Gabbett TJ. The training—injury prevention paradox: should athletes be training smarter and harder? Br J Sports Med. (2016) 50:273–80. doi: 10.1136/bjsports-2015-095788, 26758673 PMC4789704

[4] Windt J Gabbett TJ. How do training and competition workloads relate to injury? The workload—injury aetiology model. Br J Sports Med. (2017) 51:428–35. doi: 10.1136/bjsports-2016-096040, 27418321

[5] Hulin BT Gabbett TJ Caputi P Lawson DW Sampson JA. Low chronic workload and the acute: chronic workload ratio are more predictive of injury than between-match recovery time: a two-season prospective cohort study in elite rugby league players. Br J Sports Med. (2016) 50:1008–12. doi: 10.1136/bjsports-2015-095364, 26851288

[6] Murray NB Gabbett TJ Townshend AD Hulin BT McLellan CP. Individual and combined effects of acute and chronic running loads on injury risk in elite Australian footballers. Scand J Med Sci Sports. (2017) 27:990–8. doi: 10.1111/sms.12719, 27418064

[7] Damji F MacDonald K Hunt MA Taunton J Scott A. Assessing acute: chronic workload ratio methodologies for the prediction of knee pain in men's elite volleyball. Transl Sports Med. (2021) 4:677–83. doi: 10.1002/tsm2.250

[8] Enright K Green M Hay G Malone JJ. Workload and injury in professional soccer players: role of injury tissue type and injury severity. Int J Sports Med. (2020) 41:89–97. doi: 10.1055/a-0997-6741, 31801172

[9] Malone S Owen A Newton M Mendes B Collins KD Gabbett TJ. The acute: chonic workload ratio in relation to injury risk in professional soccer. J Sci Med Sport. (2017) 20:561–5. doi: 10.1016/j.jsams.2016.10.014, 27856198

[10] Carbone L Sampietro M Cicognini A García-Sillero M Vargas-Molina S. Is the relationship between acute and chronic workload a valid predictive injury tool? A Bayesian analysis. J Clin Med. (2022) 11:5945. doi: 10.3390/jcm11195945, 36233815 PMC9572878

[11] Bakal DR Friedrich TR Keane G White B Roh EY. Team's average acute: chronic workload ratio correlates with injury risk in NCAA men's soccer team. Pm&R. (2023) 15:1140–9. doi: 10.1002/pmrj.12923, 36411734

[12] Maupin D Schram B Canetti E Orr R. The relationship between acute: chronic workload ratios and injury risk in sports: a systematic review. Open Access J Sports Med. (2020) 11:51–75. doi: 10.2147/OAJSM.S231405, 32158285 PMC7047972

[13] Edwards T Spiteri T Piggott B Bonhotal J Haff GG Joyce C. Monitoring and managing fatigue in basketball. Sports. (2018) 6:19. doi: 10.3390/sports6010019, 29910323 PMC5969183

[14] Pérez-Ifrán P Rial M Brini S Calleja-González J Del Rosso S Boullosa D . Change of direction performance and its physical determinants among young basketball male players. J Hum Kinet. (2023) 85:23–34. doi: 10.2478/hukin-2022-0107, 36643832 PMC9808810

[15] McCall A Dupont G Ekstrand J. Internal workload and non-contact injury: a one-season study of five teams from the UEFA elite Club injury study. Br J Sports Med. (2018) 52:1517–22. doi: 10.1136/bjsports-2017-098473, 29626055

[16] Jones CM Griffiths PC Mellalieu SD. Training load and fatigue marker associations with injury and illness: a systematic review of longitudinal studies. Sports Med. (2017) 47:943–74. doi: 10.1007/s40279-016-0619-5, 27677917 PMC5394138

[17] Timoteo TF Debien PB Fonseca DS Felício DC Bara Filho MG. Training load and injuries in volleyball: an approach based on different methods of calculating acute to chronic workload ratio. Sports Health. (2025) 17:104–10. doi: 10.1177/19417381241293771, 39535079 PMC11561937

[18] Page MJ McKenzie JE Bossuyt PM Boutron I Hoffmann TC Mulrow CD . The PRISMA 2020 statement: an updated guideline for reporting systematic reviews. BMJ. (2021) 372:n71. doi: 10.1136/bmj.n71 PMC8005924 33782057

[19] Rico-González M Pino-Ortega J Clemente FM Los Arcos A. Guidelines for performing systematic reviews in sports science. Biol Sport. (2022) 39:463–71. doi: 10.5114/biolsport.2022.106386, 35309539 PMC8919872

[20] Bergeron MF Mountjoy M Armstrong N Chia M Côté J Emery CA . International Olympic Committee consensus statement on youth athletic development. Br J Sports Med. (2015) 49:843–51. doi: 10.1136/bjsports-2015-094962, 26084524

[21] McKay AKA Stellingwerff T Smith ES Martin DT Mujika I Goosey-Tolfrey VL . Defining training and performance caliber: a participant classification framework. Int J Sports Physiol Perform. (2022) 17:317–31. doi: 10.1123/ijspp.2021-0451 34965513

[22] Cheung MW-L. Modeling dependent effect sizes with three-level meta-analyses: a structural equation modeling approach. Psychol Methods. (2014) 19:211–29. doi: 10.1037/a0032968 23834422

[23] Assink M Wibbelink CJM. Fitting three-level meta-analytic models in R: a step-by-step tutorial. Quant Methods Psychol. (2016) 12:154–74. doi: 10.20982/tqmp.12.3.p154

[24] Sterne JA Hernán MA Reeves BC Savović J Berkman ND Viswanathan M . ROBINS-I: a tool for assessing risk of bias in non-randomised studies of interventions. BMJ. (2016) 355:i4919. doi: 10.1136/bmj.i4919, 27733354 PMC5062054

[25] Michailidis Y. A systematic review on utilizing the acute to chronic workload ratio for injury prevention among professional soccer players. Appl Sci (Basel). (2024) 14:4449. doi: 10.3390/app14114449

[26] Sánchez-Meca J Marín-Martínez F Chacón-Moscoso S. Effect-size indices for dichotomized outcomes in meta-analysis. Psychol Methods. (2003) 8:448–67. doi: 10.1037/1082-989X.8.4.448, 14664682

[27] Borenstein M Hedges LV Higgins JPT Rothstein HR. A basic introduction to fixed-effect and random-effects models for meta-analysis. Res Synth Methods. (2010) 1:97–111. doi: 10.1002/jrsm.12, 26061376

[28] Higgins JPT. Green S, editors (2011). Cochrane handbook for systematic reviews of interventions. Version 5.1. 0 (updated march 2011). The Cochrane collaboration. Available online at: https://www.cochrane.org/authors/handbooks-and-manuals/handbook/archive/v5.1.0 (Accessed August 6, 2026).

[29] Viechtbauer W. Conducting meta-analyses in R with the metafor package. J Stat Softw. (2010) 36:1–48. doi: 10.18637/jss.v036.i03

[30] Borenstein M Hedges LV Higgins JPT Rothstein HR. Introduction to meta-Analysis. 2nd ed. Hoboken, NJ: John wiley & sons (2021).

[31] Peters JL Sutton AJ Jones DR Abrams KR Rushton L. Contour-enhanced meta-analysis funnel plots help distinguish publication bias from other causes of asymmetry. J Clin Epidemiol. (2008) 61:991–6. doi: 10.1016/j.jclinepi.2007.11.010, 18538991

[32] Duval S Tweedie R. A nonparametric “trim and fill” method of accounting for publication bias in meta-analysis. J Am Stat Assoc. (2000) 95:89–98. doi: 10.1080/01621459.2000.10473905

[33] Arazi H Asadi A Khalkhali F Boullosa D Hackney AC Granacher U . Association between the acute to chronic workload ratio and injury occurrence in young male team soccer players: a preliminary study. Front Physiol. (2020) 11:608. doi: 10.3389/fphys.2020.00608, 32670083 PMC7327085

[34] Bowen L Gross AS Gimpel M Bruce-Low S Li FX. Spikes in acute: chronic workload ratio (ACWR) associated with a 5–7 times greater injury rate in English premier league football players: a comprehensive 3-year study. Br J Sports Med. (2020) 54:731–8. doi: 10.1136/bjsports-2018-099422, 30792258 PMC7285788

[35] Colby MJ Dawson B Peeling P Heasman J Rogalski B Drew MK . Multivariate modelling of subjective and objective monitoring data improve the detection of non-contact injury risk in elite Australian footballers. J Sci Med Sport. (2017) 20:1068–74. doi: 10.1016/j.jsams.2017.05.010, 28595869

[36] Delecroix B McCall A Dawson B Berthoin S Dupont G. Workload and non-contact injury incidence in elite football players competing in European leagues. Eur J Sport Sci. (2018) 18:1280–7. doi: 10.1080/17461391.2018.1477994, 29860935

[37] Esmaeili A Hopkins WG Stewart AM Elias GP Lazarus BH Aughey RJ. The individual and combined effects of multiple factors on the risk of soft tissue non-contact injuries in elite team sport athletes. Front Physiol. (2018) 9:1280. doi: 10.3389/fphys.2018.01280, 30333756 PMC6176657

[38] Hulin BT Gabbett TJ Blanch P Chapman P Bailey D Orchard JW. Spikes in acute workload are associated with increased injury risk in elite cricket fast bowlers. Br J Sports Med. (2014) 48:708–12. doi: 10.1136/bjsports-2013-092524, 23962877

[39] Jaspers A Kuyvenhoven JP Staes F Frencken WGP Helsen WF Brink MS. Examination of the external and internal load indicators’ association with overuse injuries in professional soccer players. J Sci Med Sport. (2018) 21:579–85. doi: 10.1016/j.jsams.2017.10.005, 29079295

[40] Murray NB Gabbett TJ Townshend AD Blanch P. Calculating acute: chronic workload ratios using exponentially weighted moving averages provides a more sensitive indicator of injury likelihood than rolling averages. Br J Sports Med. (2017) 51:749–54. doi: 10.1136/bjsports-2016-097152, 28003238

[41] Pajuelo A Caparrós T. Acute: chronic workload ratio. Exploration and applicability in women's amateur football. Apunts Educ Fis Deport. (2021) 145:25–32. doi: 10.5672/apunts.2014-0983.es.(2021/3).145.04

[42] Sampson JA Murray A Williams S Halseth T Hanisch J Golden G . Injury risk-workload associations in NCAA American college football. J Sci Med Sport. (2018) 21:1215–20. doi: 10.1016/j.jsams.2018.05.019, 29843960

[43] Iwasaki Y Someya Y Nagao M Nozu S Shiota Y Takazawa Y. Relationship between the contact load and time-loss injuries in rugby union. Front. Sports Active Liv. (2024) 6:1395138. doi: 10.3389/fspor.2024.1395138, 39398271 PMC11466806

[44] Nobari H Alijanpour N Martins AD Oliveira R. Acute and chronic workload ratios of perceived exertion, global positioning system, and running-based variables between starters and non-starters: a male professional team study. Front Psychol. (2022) 13:860888. doi: 10.3389/fpsyg.2022.860888, 35369219 PMC8970311

[45] Sampson JA Murray A Williams S Sullivan A Fullagar HHK. Subjective wellness, acute: chronic workloads, and injury risk in college football. J Strength Cond Res. (2019) 33:3367–73. doi: 10.1519/JSC.0000000000003000, 30747901

[46] Seco-Serna R Lago-Fuentes C Barcala-Furelos M. The acute: chronic workload ratio and injury risk in semiprofessional football players. Int J Sports Med. (2024) 45:684–9. doi: 10.1055/a-2282-0024, 38442910

[47] Tiernan C Comyns T Lyons M Nevill AM Warrington G. The association between training load indices and injuries in elite soccer players. J Strength Cond Res. (2022) 36:3143–50. doi: 10.1519/JSC.0000000000003914, 33298712

[48] Veiga G Torres G Maposa I. Association of the acute: chronic workload ratio and wellness scores in premier league male hockey players. S Afr J Sports Med. (2021) 33. doi: 10.17159/2078-516X/2021/v33i1a9244, 36816909 PMC9924552

[49] Wu J Zhao F Li C. Analyzing activity and injury risk in elite curling athletes: seven workload monitoring metrics from session-RPE. Front Public Health. (2024) 12:1409198. doi: 10.3389/fpubh.2024.1409198, 39193197 PMC11347442

[50] Nobari H Alves AR Haghighi H Clemente FM Carlos-Vivas J Pérez-Gómez J . Association between training load and well-being measures in young soccer players during a season. Int J Environ Res Public Health. (2021) 18:4451. doi: 10.3390/ijerph18094451, 33922250 PMC8122726

[51] Cummins C Welch M Inkster B Cupples B Weaving D Jones B . Modelling the relationships between volume, intensity and injury-risk in professional rugby league players. J Sci Med Sport. (2019) 22:653–60. doi: 10.1016/j.jsams.2018.11.028, 30651223

[52] Fanchini M Rampinini E Riggio M Coutts AJ Pecci C McCall A. Despite association, the acute: chronic work load ratio does not predict non-contact injury in elite footballers. Sci Med Footb. (2018) 2:108–14. doi: 10.1080/24733938.2018.1429014

[53] Malone S Owen A Mendes B Hughes B Collins K Gabbett TJ. High-speed running and sprinting as an injury risk factor in soccer: can well-developed physical qualities reduce the risk? J Sci Med Sport. (2018) 21:257–62. doi: 10.1016/j.jsams.2017.05.016, 28595870

[54] Mohr P Matias T de Lucas R. Association between internal training load and muscle injuries in Brazilian professional soccer players. Biol Sport. (2023) 40:675–9. doi: 10.5114/biolsport.2023.119285, 37398960 PMC10286624

[55] Stares J Dawson B Peeling P Heasman J Rogalski B Drew M . Identifying high risk loading conditions for in-season injury in elite Australian football players. J Sci Med Sport. (2018) 21:46–51. doi: 10.1016/j.jsams.2017.05.012, 28601588

[56] Timoteo TF Debien PB Miloski B Werneck FZ Gabbett T Bara Filho MG. Influence of workload and recovery on injuries in elite male volleyball players. J Strength Cond Res. (2021) 35:791–6. doi: 10.1519/JSC.0000000000002754, 30113918

[57] Weiss KJ Allen SV McGuigan MR Whatman CS. The relationship between training load and injury in men’s professional basketball. Int J Sports Physiol Perform. (2017) 12:1238–42. doi: 10.1123/ijspp.2016-0726, 28253031

[58] Malone S Roe M Doran DA Gabbett TJ Collins K. High chronic training loads and exposure to bouts of maximal velocity running reduce injury risk in elite Gaelic football. J Sci Med Sport. (2017) 20:250–4. doi: 10.1016/j.jsams.2016.08.005, 27554923

[59] Cross MJ Williams S Trewartha G Kemp SPT Stokes KA. The influence of in-season training loads on injury risk in professional rugby union. Int J Sports Physiol Perform. (2016) 11:350–5. doi: 10.1123/ijspp.2015-0187, 26309331

[60] Oliveira R Martins A Nobari H Nalha M Mendes B Clemente FM . In-season monotony, strain and acute/chronic workload of perceived exertion, global positioning system running based variables between player positions of a top elite soccer team. BMC Sports Sci Med Rehabil. (2021) 13:126. doi: 10.1186/s13102-021-00356-3, 34641968 PMC8620662

[61] West SW Williams S Cazzola D Kemp S Cross MJ Stokes KA. Training load and injury risk in elite rugby union: the largest investigation to date. Int J Sports Med. (2021) 42:731–9. doi: 10.1055/a-1300-2703, 33291182

[62] Bowen L Gross AS Gimpel M Li F-X. Accumulated workloads and the acute: chronic workload ratio relate to injury risk in elite youth football players. Br J Sports Med. (2017) 51:452–9. doi: 10.1136/bjsports-2015-095820, 27450360 PMC5460663

[63] Carey DL Blanch P Ong K-L Crossley KM Crow J Morris ME. Training loads and injury risk in Australian football—differing acute: chronic workload ratios influence match injury risk. Br J Sports Med. (2017) 51:1215–20. doi: 10.1136/bjsports-2016-096309, 27789430 PMC5537557

[64] Fousekis A Fousekis K Fousekis G Vaitsis N Terzidis I Christoulas K . Two or four weeks acute: chronic workload ratio is more useful to prevent injuries in soccer? Appl Sci. (2023) 13:495. doi: 10.3390/app13010495

[65] Malone S Roe M Doran DA Gabbett TJ Collins KD. Protection against spikes in workload with aerobic fitness and playing experience: the role of the acute: chronic workload ratio on injury risk in elite Gaelic football. Int J Sports Physiol Perform. (2017) 12:393–401. doi: 10.1123/ijspp.2016-0090, 27400233

[66] Haddad M Stylianides G Djaoui L Dellal A Chamari K. Session-RPE method for training load monitoring: validity, ecological usefulness, and influencing factors. Front Neurosci. (2017) 11:612. doi: 10.3389/fnins.2017.00612, 29163016 PMC5673663

[67] Scott TJ Black CR Quinn J Coutts AJ. Validity and reliability of the session-RPE method for quantifying training in Australian football: a comparison of the CR10 and CR100 scales. J Strength Cond Res. (2013) 27:270–6. doi: 10.1519/JSC.0b013e3182541d2e, 22450253

[68] Roell M Roecker K Gehring D Mahler H Gollhofer A. Player monitoring in indoor team sports: concurrent validity of inertial measurement units to quantify average and peak acceleration values. Front Physiol. (2018) 9:141. doi: 10.3389/fphys.2018.00141, 29535641 PMC5835232

[69] Torres-Ronda L Beanland E Whitehead S Sweeting A Clubb J. Tracking systems in team sports: a narrative review of applications of the data and sport specific analysis. Sports Med. Open. (2022) 8:15. doi: 10.1186/s40798-022-00408-z, 35076796 PMC8789973

[70] Memmert D Lemmink KA Sampaio J. Current approaches to tactical performance analyses in soccer using position data. Sports Med. (2017) 47:1–10. doi: 10.1007/s40279-016-0562-5, 27251334

[71] Folgado H Gonçalves B Sampaio J. Positional synchronization affects physical and physiological responses to preseason in professional football (soccer). Res Sports Med. (2018) 26:51–63. doi: 10.1080/15438627.2017.1393754, 29058465

[72] Wang C Vargas JT Stokes T Steele R Shrier I. The acute: chronic workload ratio: challenges and prospects for improvement. arXiv. [Preprint]. (2019) arXiv:1907.05326.

