# Supplementary material for: Acute:chronic workload ratio and load management for team sports: a multilevel meta-analysis
Source: Front Public Health. 2026 Aug 13;14:1896651. doi: 10.3389/fpubh.2026.1896651 (PMC13520966; doi:10.3389/fpubh.2026.1896651)
Supplement: Supplementary file 1 [file Data_Sheet_1.PDF]

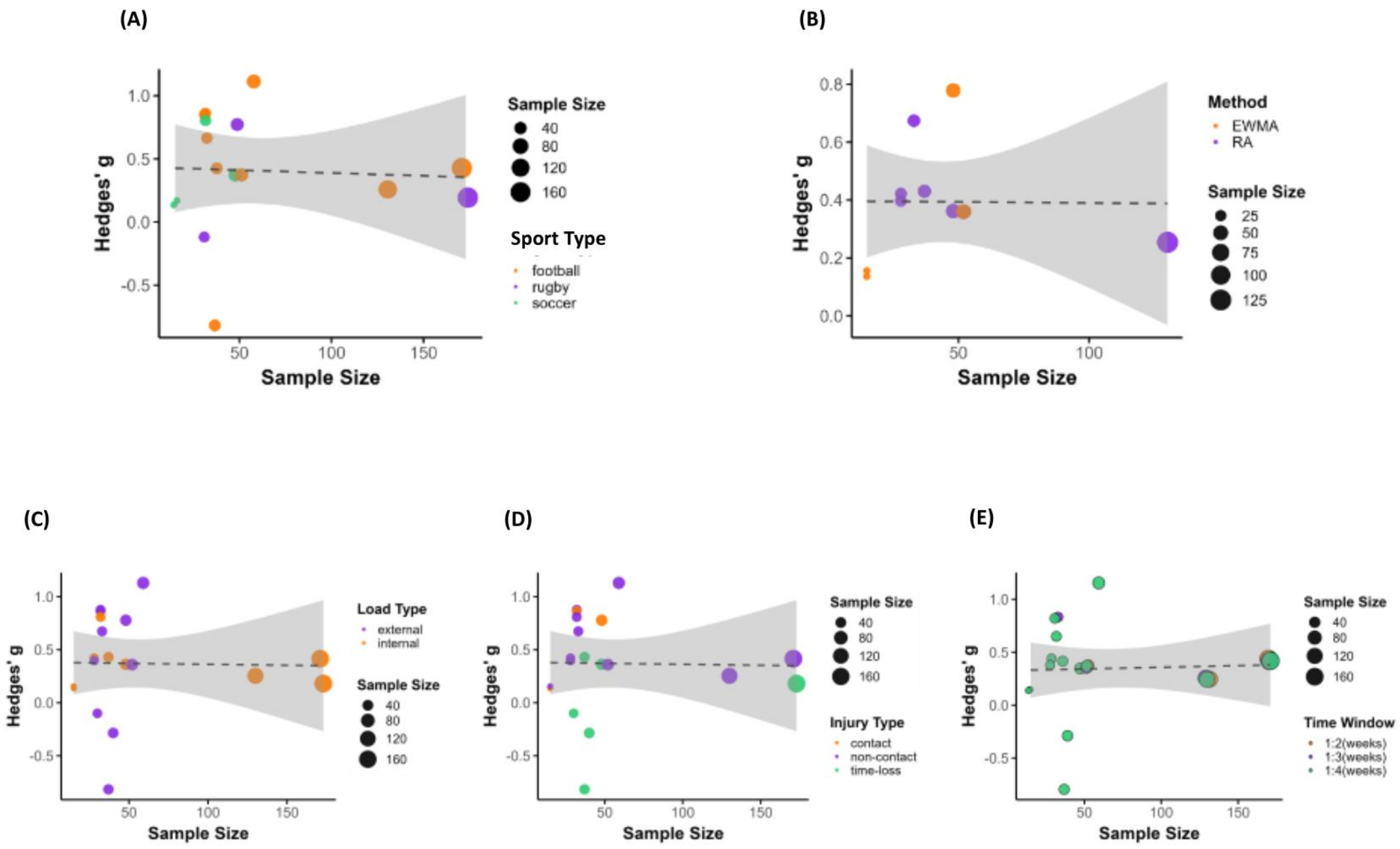

Figure S1: Bubble plots of meta-regression analyses examining the moderating effects of sport type (A), ACWR calculation method (B), load type (C), injury type (D), and time window (E) on the relationship between ACWR and injury risk.

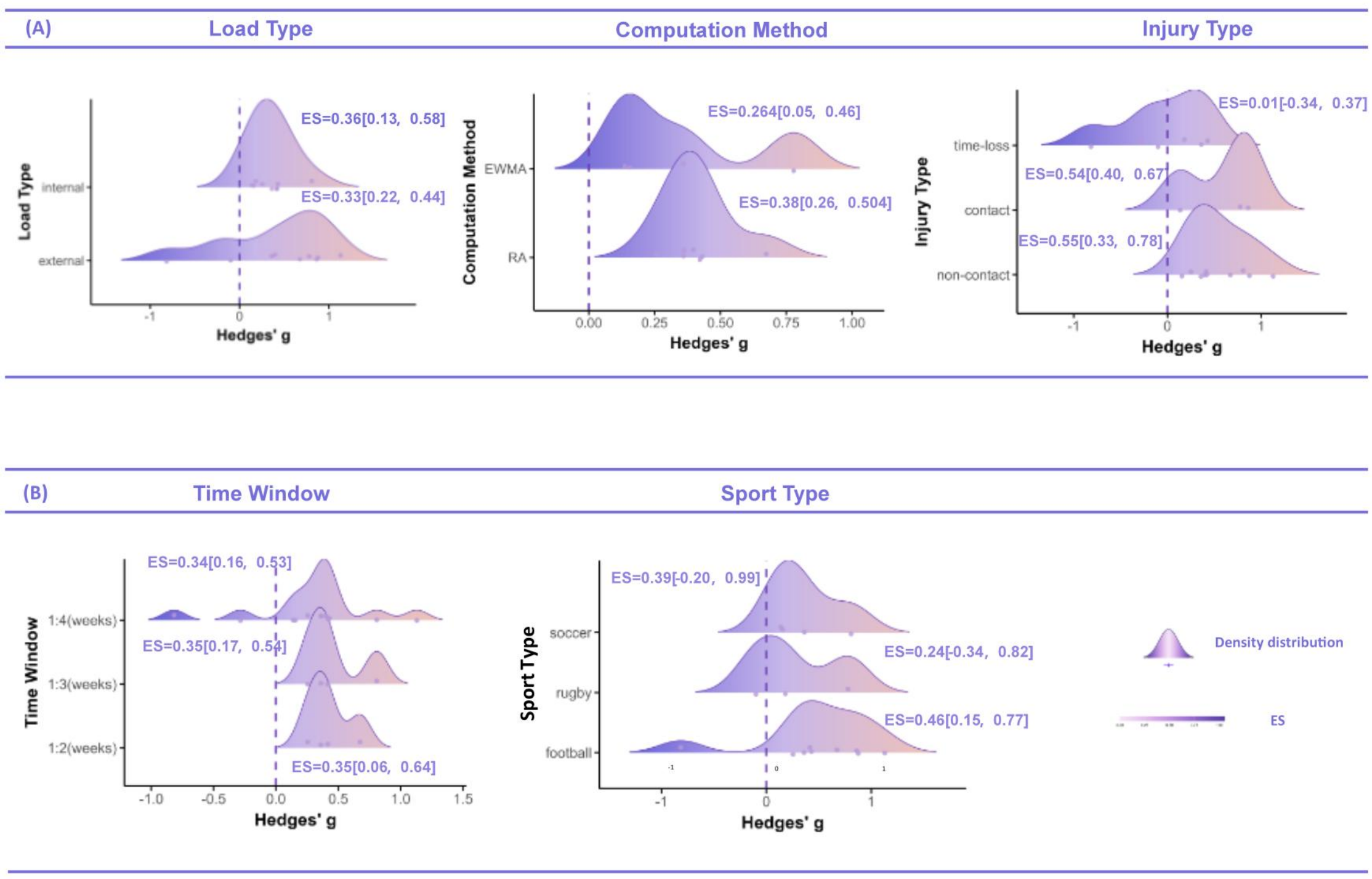

Figure S2: Ridge plots of subgroup moderator effects in the multilevel meta-analysis of ACWR and injury risk.

Table S1. Inclusion and exclusion criteria based on the PICOS framework

PICOS	Inclusion criteria	Exclusion criteria
Population	Competitive team-sport athletes at professional, semi-professional, national, university, or elite-youth levels. Elite-youth athletes were eligible when participating in structured high-performance pathways or equivalent competitive contexts.	Athletes participating in individual sports; recreational participants.
Intervention/exposure	ACWR derived from internal, external, or combined training- or competition-load measures; ACWR values reported or derivable.	Workload ratios other than ACWR; ACWR values neither reported nor derivable.
Comparator	Any ACWR exposure comparison or statistical contrast permitting evaluation of the association between ACWR and injury, including ACWR categories,	No eligible ACWR-based comparison, contrast, or association.

	continuous estimates, or injured-versus-uninjured comparisons.	
Outcomes	Injury incidence or injury risk, with an association between ACWR and injury outcomes reported or derivable.	No injury-related outcome or no evaluation of the ACWR-injury association.
Study design	Prospective or retrospective cohort studies, randomized controlled trials, and other longitudinal observational studies.	Narrative reviews, conference abstracts, editorials, and case reports; non-longitudinal designs not evaluating the ACWR-injury relationship.

Table S2: Formulae for RA and EWMA models

Model Type	Calculation Formula
Rolling Average (RA)	RA = (Load_week1 + Load_week2 + Load_week3 + Load_week4) / 4
Exponentially Weighted Moving Average (EWMA)	EWMA_today = (Load_today * λ) + ((1 – λ) * EWMA_yesterday)

*λ= 2/(N+1), N = time period (e.g., 28 days)

Table S3: Comparison of Coupled and Uncoupled Calculation Approaches

	Coupled	Uncoupled
Equation Example	$\frac{A}{0.25*(W1+W2+W3+A)}$	$\frac{A}{0.333*(W2+W3+W4)}$
Definition	Ratio of the most recent week's load (A) to the average load of the previous four weeks (W1-W4, where W1 is the week immediately preceding A)	Ratio of the most recent week's load (A) to the average load of the three weeks preceding it (W2-W4)

Note: 'A' denotes acute load; W1, W2, W3, W4 represent weekly loads chronologically, with W1 being the most recent week before the acute week.

Table S4: Summary of Moderator Effects in Multilevel Meta-Analysis

Moderator	τ^2 (Level 2)	τ^2 (Level 1)	QE (df)	QE p	QM (df)	QM p
Load type	0.173	0	260.36 (17)	＜0.0001	0.200 (1)	0.655
Calculation method	0.169	0	177.13（18）	＜0.0001	1.27（1）	0.25
Sport type	0.1694	0	102.59（13）	＜0.0001	0.55（2）	0.7605
Time window	0.1685	0	177.13（18）	＜0.0001	0.009（2）	0.996
Injury type	0.1022	0	159.54	＜0.0001	8.66（2）	0.0132

(16)

Table S5: Databases Searched and Search Terms Used in the Literature Review

Databases	Search Strategy
Web of Science	TS=("acute:chronic workload ratio" OR "ACWR") AND TS=("injury" OR "pain") AND TS=("load management" OR "training load") AND TS=("team sport" OR "soccer" OR "basketball" OR "rugby")
PubMed	("acute:chronic workload ratio"[Title/Abstract] OR "ACWR"[Title/Abstract]) AND ("injury"[Title/Abstract] OR "injuries"[Title/Abstract] OR "pain"[Title/Abstract]) AND ("load management"[Title/Abstract] OR "training load"[Title/Abstract]) AND ("team sport"[Title/Abstract] OR "soccer"[Title/Abstract] OR "basketball"[Title/Abstract] OR "rugby"[Title/Abstract])
Scopus	TITLE-ABS-KEY("acute:chronic workload ratio" OR "ACWR") AND TITLE-ABS-KEY("injury" OR "pain") AND TITLE-ABS-KEY("load management" OR "training load") AND TITLE-ABS-KEY("team sport" OR "soccer" OR "basketball" OR "rugby")
SPORTDiscus (via EBSCOhost)	TX ("acute:chronic workload ratio" OR "ACWR") AND TX ("injury" OR "pain") AND TX ("load management" OR "training load") AND TX ("team sport" OR "soccer" OR "basketball" OR "rugby")

Table S6. Detailed Exclusion Rationale for Studies Not Included in the Meta-Analysis

Author(Year)	Reason for Exclusion	Explanation
Arazi_2020	No extractable effect size	Only reported correlation coefficient (r) between ACWR and injury, no OR/RR or group-based injury risk provided
Carey_2017	No extractable effect size	RR values were model-derived and not based on raw injury event data; insufficient information for meta-analytic conversion
Colby_2017	Incompatible outcome metric	Only adjusted IRRs from multivariate models were reported; no raw effect size or group-level

		data provided for meta-analysis
Cummins_2018	No extractable effect size	Only injury incidence rates and z-score group comparisons reported; no OR/RR or raw injury data provided
Damji_2020	No extractable effect size	Used group comparisons and t-tests (e.g., Cohen's d); no OR/RR or event rates reported
Enright_2019	No extractable effect size	Reported injury percentage across ACWR levels, but no OR/RR or raw event counts provided
Esmaeili_2018	No extractable effect size	Investigated illness burden, not injury risk; no OR/RR or injury-related effect estimates were reported
Fanchini_2018	No extractable effect size	Reported group differences and Cohen's d, but no OR/RR or raw injury counts suitable for effect size computation
Fousekis_2023	No extractable effect size	Used non-parametric comparisons across ACWR levels without reporting OR/RR or regression-based injury risk estimates
Jaspers_2017	No extractable effect size	Reported overuse injuries only; no OR/RR or effect estimates suitable for analysis of overall or acute injury risk
Hulin_2016a	No extractable effect size	Reported ACWR–injury relationship at the team level; no individual-level effect sizes or event counts were available
Hulin_2016b	No extractable effect size	Analyzed same cohort as Hulin et al. (2014); focused only on non-contact lower limb soft-tissue injuries, reducing generalizability.
Murray_2016	Incompatible outcome metric	Although injury likelihood and RR were reported, effect estimates were presented across multiple ACWR ranges without sufficient raw event counts or standard errors to allow consistent extraction or conversion for multilevel meta-analysis
Nobari_2021	Incompatible outcome metric	Investigated associations between

		ACWR and well-being measures (e.g., fatigue, stress, DOMS), but did not report injury incidence or injury-related risk estimates
Nobari_2022	Incompatible outcome metric	Focused on seasonal variations and group differences (starters vs. non-starters) in ACWR and EWMA metrics; no injury incidence or ACWR–injury association was analyzed
Oliveira_2021	Incompatible outcome metric	Examined positional differences in ACWR, training monotony, and strain; injury occurrence or injury risk was not assessed as an outcome
Pajuelo_2021	No extractable effect size	Although injury rate differences across ACWR/EWMA categories were reported, no OR/RR, confidence intervals, or raw injury counts were provided to compute extractable effect sizes
Sampson_2019	Incompatible outcome metric	Although relative risks (RR) were reported for injury at extreme ACWR values, the analyses primarily focused on interaction effects between ACWR and subjective wellness states; effect estimates were presented across multiple SD-based ACWR categories without sufficient raw event counts or standard errors for consistent extraction in multilevel meta-analysis
Seco-Serna_2024	No extractable effect size	Examined the association between ACWR and non-contact injuries using Spearman correlations and diagnostic accuracy metrics (sensitivity/specificity), but did not report OR/RR, hazard ratios, or regression-based effect estimates suitable for meta-analysis
Stares_2017	Incompatible outcome metric	Reported incidence rate ratios (IRR) across numerous ACWR × chronic load combinations derived from the same cohort; effect estimates were

		condition-specific and model-dependent, without a single extractable ACWR–injury contrast suitable for standardized multilevel meta-analytic pooling
Timoteo_2018	No extractable effect size	Compared ACWR values between injured and uninjured players and reported group differences and logistic regression significance, but did not provide OR/RR with confidence intervals or raw injury counts stratified by ACWR exposure levels
Wu_2024	No extractable effect size	Study focused on elite curling athletes, which is not a typical team ball sport; small sample size (n = 8); although injuries were recorded, no individual-level ACWR–injury associations or effect estimates were reported
Weiss_2017	No extractable effect size	Injury proxy was based on coach-reported time-loss but lacked clinical validation; no raw injury counts, OR/RR values, or regression estimates were provided across ACWR ranges
West_2021	Incompatible outcome metric	Study modeled performance-based fatigue risk from ACWR, without collecting or analyzing actual injury incidence; no injury-related outcome was defined or assessed
Veiga_2021	Incompatible outcome metric	Reported "physical problem" occurrence descriptively without standardized injury definition, count, or severity classification; lacked effect size estimates or data suitable for meta-analysis extraction

Table S7. Original Effect Measures (OR/RR) and Corresponding Hedge' g Values Used in the Meta-analysis

Study_id	Effect_size	ci_lower	ci_upper	metric_type	g	95%CI
Bowen2017	5.11	1.75	14.96	RR	0.88	[0.30,1.46]
Bowen2017	4.98	1.31	19.02	RR	0.86	[0.14,1.59]
Bowen2020	3.5	1.5	8.2	RR	0.68	[0.22,1.13]

Cross2015	1.39	0.98	1.98	OR	0.18	[-0.01,0.37]
Delecroix2018	1.59	1.18	2.15	RR	0.25	[0.09,0.42]
Hulin2014	2.2	1.91	2.53	RR	0.42	[0.35,0.50]
Hulin2014	2.1	1.81	2.44	RR	0.40	[0.32,0.48]
Iwasaki2024	4.2	1.74	10.11	OR	0.78	[0.30,1.26]
Malone2016a	2.22	1.45	3.36	OR	0.43	[0.20,0.66]
Malone2016b	1.95	0.98	3.95	OR	0.36	[-0.02,0.74]
Malone2017a	0.22	0.10	1.22	OR	-0.82	[-1.39,-0.25]
Malone2018	0.59	0.37	0.82	OR	-0.29	[-0.50,-0.07]
McCall2017	2.13	1.21	3.77	RR	0.42	[0.10,0.73]
Mohr2023	4.5	1.5	13.3	OR	0.81	[0.22,1.40]
Murray2016a	7.98	5.86	10.88	RR	1.13	[0.96,1.30]
Sampson2018	1.94	1.07	3.52	RR	0.36	[0.04,0.68]
Tiernan2022	1.3	1.05	1.59	OR	0.14	[0.03,0.24]
Tiernan2022	1.35	1.14	1.59	OR	0.16	[0.07,0.24]
Windt2016	0.83	0.7	0.99	OR	-0.10	[-0.19,-0.01]

Table S8. Practical framework for interpreting ACWR in athlete monitoring.

Decision step	Key question	Interpretation and recommended action
1. Confirm data adequacy	Are workload data collected consistently, and is the athlete’s recent load history sufficiently complete and stable?	If yes, examine the within-athlete trend. If data are incomplete, the monitoring method has changed, or chronic-load history is unstable (for example, early preseason or RTP), do not interpret ACWR as a reliable injury-risk signal.
2. Identify a workload deviation	Does ACWR differ meaningfully from the athlete’s recent progression or individual reference pattern?	Use the deviation as a flag for further review, not as an injury probability or diagnosis. Confirm data quality and avoid interpreting a single value in isolation.
3. Establish context	Could the change reflect competition congestion, season phase, position-specific demands, planned overload, detraining, or RTP?	Interpret ACWR in relation to the athlete’s current sporting and clinical context. Do not apply a universal “safe” or “high-risk” threshold.
4. Review complementary indicators	Are symptoms, wellness, recovery, previous injury, neuromuscular function, and physiological or biomechanical indicators consistent with the workload signal?	Concern is strengthened when several indicators converge. An isolated ACWR deviation without other concerns should not automatically lead to training restriction.
5. Decide and reassess	Is there convergent evidence of reduced readiness or clinical concern?	Consider closer assessment, recovery support, or an individualized training adjustment. Do not use ACWR alone to determine whether an athlete should train, compete, or return to play;

	document the decision and reassess longitudinally.

*: ACWR, acute: chronic workload ratio; RTP: return to play

Table S9：Basic Characteristics of Included Studies

Reference	Height (cm)	Weight (kg)	Sex	Age (years)	N	Sport type	Level	Workload Metric	Time Window	Calculation Model	Main Findings
Arazi2020	175.4 ± 5.6	76.3 ± 6.4	-	17.1±0.7	22	Soccer	Semi-professional	sRPE	-	EWMA, RA	Non-contact injuries were significantly more frequent than contact injuries, and there was a small-to-moderate correlation between ACWR and non-contact injuries. This study found that the acute:chronic workload ratio calculated using the EWMA model showed a stronger association with injury incidence in male players than the rolling average model.
Bowen2017	180.0±7.3	74.1±7.0	-	17.3±0.9	32	Football	Elite youth	GPS	-	-	Higher cumulative and acute training loads are associated with an increased risk of injury. A progressive increase in chronic load may

											enhance tolerance to higher acute loads and improve resistance to injury.
Bowen2020	182.0±6.9	79.9±7.7	-	25.4±3.1	33	Football	Professional	GPS	1:4 (weeks)	RA	Among athletes in the English Football League, the relationship between ACWR and non-contact injury risk was stronger than that of cumulative load, suggesting that rapid increases in load are more predictive of injury than cumulative load.
Carey2017	188.2±6.7	85.7±8.1	-	22.9±4.0	53	Football	Professional	GPS	-	-	The results indicate that the optimal time window for calculating the ACWR may need to be determined according to different sports or teams and may depend on the specific scheduling of

											athletes' matches and training.
Colby2017	188.1±6.6	87.0±8.2	-	22.9±3.4	70	Football	Professional	sRPE, GPS	1:4 (weeks)	RA	Compared with the past 21 days, the proportion of moderate-speed running volume over the past 3 days best explained the likelihood of injury during competition. Low chronic load combined with large acute load increases or peak loads should be regarded as potential injury risk factors. Chronic load is an important moderator of the relationship between training load and injury, with low chronic load combined with either low or very high ACWR being associated with an increased risk of injury.

Cross2015	-	-	-	-	173	Rugby	Professional	sRPE	-	-	Players' weekly and four-week cumulative loads should be monitored, and the use of alternating high- and low-intensity training weeks as a periodization strategy is not recommended.
Cummins2018	-	-	Male	-	48	Rugby	Professional	GPS	1:4 (weeks)	-	As duration, total distance, and player load increase, the risk of injury also increases. Conversely, with four training sessions per week, increases in total distance, accelerations, decelerations, and player load are associated with a reduced risk of injury.
Damji2021	197.3±6.4	92.7±7.0	Male	-	12	Volleyball	Elite youth	-	1:4 (weeks)	EWMA, Coupled, Uncoupled	The coefficient estimate for ACWR variability was small and not statistically significant, indicating

											that the variability used had little impact on the relationship with knee pain scores.
Delecroix2018	-	-	-	-	130	Football	Professional	sRPE	4 weeks, 3 weeks, 2 weeks, 1 weel	RA	When the cumulative four-week absolute load exceeded 10,629 arbitrary units, the relative risk of non-contact injury increased, whereas no such effect was observed when the load ranged from 3,745 to 10,628 AU.
Enright2019	-	-	-	-	192	Soccer	Professional	GPS	4 weeks, 3 weeks, 2 weeks, 1 week	EWMA, Coupled, Uncoupled	There were no differences in cumulative weekly load or ACWR calculations between muscle, ligament, and tendon injuries. This study shows that, with the current variables, cumulative weekly load or the ACWR method

											has limited ability to differentiate between injury types and severities.
Esmaeili2018	-	-	Male	22.9±3.9	55	Football	Professional	sRPE, GPS	-	RA, EWMA	High GPS/accelerometer-derived ACWRs showed stronger effects after adjusting for previous leg injuries. This unexpected finding may be due to the inability of GPS/accelerometer devices to capture most of the training performed by injured players during rehabilitation, resulting in inconsistent ACWRs at the early stages of full training resumption.
Fanchini2018	182±5	78±4	-	26±5	34	Football	Professional	RPE	1:2 (weeks), 1:3	-	There is an association between acute and chronic markers, but

									(weeks), 1:4 (weeks)		their predictive ability is poor.
Fousekis2023	180.4±2.6	76.2±3.8	-	21.1±0.6	35	Soccer	Professional	GPS	-	-	Professional football players may sustain non-contact injuries. Observations indicated that the ACWR2 model may be a more sensitive indicator, as differences were more pronounced across a greater number of examined variables. We recommend using the ACWR2 model because it provides more immediate feedback.
Hulin2014	-	-	-	26±5	28	Cricket	Elite youth	Weekly Pitch Count, sRPE	1:4 (weeks)	RA	These findings suggest that large increases in acute training load are associated with an increased risk of injury in elite fast bowlers in cricket.
Hulin2016a	184.5±4.9	99.5±8.0	-	24.8±3.4	28	Rugby	Elite youth	GPS	1:4 (weeks)	RA	This study suggests that

										low chronic workload and the acute-to-chronic workload ratio are better predictors of injury risk than recovery time alone. Specifically, higher chronic workloads can buffer acute workload spikes, which were associated with the greatest risk of match injuries in this study.
Hulin2016b	-	-	-	23.4±3.5	53	Rugby	Elite youth	GPS	1:4 (weeks)	-	This study demonstrated an association between simple distance measures and injury risk in elite rugby players.
Iwasaki2024	180.7±7.6	97.2±11.8	Male	27.5±3.1	48	Rugby	Elite youth	GPS	-	EWMA	The RA-ACWR essentially has weak predictive ability and introduces statistical artifacts.

Jaspers2017	182±6	77.5±7.4	Male	23.2±3.7	35	Soccer	Professional	sRPE, GPS	1:4 (weeks)	RA	The study results indicate that changes in injury risk are primarily associated with external load metrics.
Malone2016a	180±7	81±7	-	24±6	37	Football	Elite youth	sRPE	1:4 (weeks)	RA	Injury risk in elite Gaelic football players is influenced by aerobic fitness, playing experience, and the ACWR
Malone2016b	183±7	72±7	-	25.3±3.1	48	Soccer	Elite youth	sRPE	1:4 (weeks)	RA	This study shows that professional football players exhibit a protective effect at ACWR between 1.00 and 1.25. When exposed to high-intensity training, athletes with greater intermittent aerobic capacity demonstrate stronger protection against injury.
Malone2017	179±5	79±7	-	24±3	37	Football	Elite youth	sRPE, GPS	1:4 (weeks)	-	Compared with the low-load group (<4750

											AU), athletes with long-term high training loads (>4750 AU) were able to better tolerate long-distance running and frequent maximal speed tests.
Malone2018	182.2±7.1	81.3±3.7	Male	26.2±4.4	40	Hurling	Amateur	sRPE	1:4 (weeks)	-	The present study found that faster players had a lower risk of injury over 5 m, 10 m, and 20 m distances.
McCall2017	181.6±6.7	77.5±7.2	-	25.1±4.9	171	Football	Elite youth	sRPE	2 weeks, 3 weeks, 4 weeks	-	The ACWR over 1:3 and 1:4 week periods was associated with non-contact injuries ($P < 0.05$).
Mohr2023	181±7	77±8	-	27.5±4.8	32	Soccer	Professional	sRPE	3 weeks, 4 weeks	-	The study found significant associations between cumulative training load and injury occurrence over three weeks (C3, $p = 0.003$) and four weeks (C4, $p = 0.023$). Athletes in the high-load group had

											a higher risk of injury compared with those in the moderate-load group.
Murray2016a	189±7	88±8	-	23±4	59	Football	Elite youth	GPS	1:4 1(weeks)	-	During the season, an ACWR of total distance greater than 2.0 was associated with a 5- to 8-fold increase in injury risk in the current and subsequent week.
Murray2016b	176.8±5.6	67.3±5.7	-	16.1±0.2	21	Soccer	Elite youth	GPS	1:4 (weeks)	RA, EWMA	Regardless of the model used, large increases in training load were associated with a higher risk of injury. However, the EWMA model was more sensitive in detecting the increase in injury risk with rising ACWR values.
Nobari2021	180±10	74.8±2.3	Male	29.4±4.4	20	Soccer	Professional	sRPE	-	Uncoupled	Health status can serve as an important indicator for coaches to

											evaluate elite athletes' training responses, providing key evidence for the development of individualized training programs.
Nobari2022	-	-	-	25.4±4.1	17	Soccer	Elite youth	sRPE、GPS	-	EWMA, Coupled, Uncoupled	s-RPE, TD, and HSRD. Both ACWR and the EWMA based on sprint distance were higher at the start of the early season than at any other time.
Oliveria2021	164.9±3.93	58.08±4.75	Female	22.87±4.8	17	Football	Amateur	sRPE、GPS	-	-	This study provides coaches and technical staff with new insights into the variability characteristics of TM, TS, and ACWR, which were calculated from internal and external load measures over 10 mesocycles across different player positions.
Pajuelo2021	187.6±8.4	103.0±20	-	20.7±1.5	52	American	Elite youth	sRPE	-	RA、	For both EL and IL, the

		.0				Football				EWMA	EWMA may be more closely related to the number of injuries than the ACWR.
Sampson2018	186.4±6.7	102.8±17.4	-	20.5±1.2	42	American Football	Elite youth	-	1:2(weeks), 1:3 (weeks), 1:4 (weeks)	RA, EWMA	The 7- to 21-day EWMA ACWR was most closely associated with a 3-day injury lag (R^2 = 0.54).
Sampson2019	-	-	Female	-	12	Volleyball	Elite youth	GPS	1:3 (weeks)	EWMA	In this study on collegiate American football, both low and high ACWR values were associated with an increased risk of injury.
Seco-Serna2024	178.48±5.05	75.83±9.86	-	25.59±4.57	17	Football	Semi-professional	sRPE	1:4 (weeks)	EWMA	There was a weak correlation between ACWR and non-contact injuries ($r = 0.069$, $p < 0.05$).
Stares2017	-	-	-	22.9±3.4	70	Football	Professional	sRPE、GPS	Acute 1-2(weeks)/ Chronic 3-8(weeks)	-	Injury risk was greatest when chronic load was low and ACWR values were either low or high.
Tiernan2022	180.8±5.8	77.1±5.1	Male	23.4±4.8	15	Soccer	Elite youth	sRPE	1:4 (weeks)	EWMA, Uncoupled	To reduce the potential risk of non-contact

											injuries, training loads should be increased gradually, avoiding weekly acute load changes (≥9%) or increases in EWMA ACWR (>1.20).
Timoteo2018	197.0±7.9	95.8±8.2	Male	26.7±5.5	14	Volleyball	Elite youth	sRPE	1:4 (weeks)	-	In elite men's volleyball, daily monitoring of workload and recovery is reasonable to reduce the likelihood of injury.
Veiga2021	174.7±6.1	72.8±8.0	Male	19-24	14	Hockey	Professional	sRPE	1:4 (weeks)	EWMA	Using subjective wellness measures as a workload management tool in hockey may have some value. Further research is needed to determine the optimal ACWR values for this sport.
Weiss2017	195.0±7.6	96.3±11.6	Male	24.4±4.7	13	Basketball	Professional	sRPE	1:4 (weeks)	-	Maintaining a training load ratio of 1 to 1.5 may be the optimal choice for professional

											basketball players' preparation.
West2021	-	-	-	-	696	Rugby	Elite youth	sRPE	-	-	In soft tissue injuries, a higher proportion of significant effects was observed with uncoupled EWMA values.
Windt2016	-	-	-	25±3	30	Rugby	Elite youth	GPS	-	-	Maximizing participation in preseason training can protect elite rugby league players from in-season injuries.
Wu2024	181.1±10.7	77.2±5.5	Male	22–31	8	Curling	Professional	sRPE	1:3 (weeks), 1:4 (weeks)	EWMA	The delayed EWMA is a more sensitive indicator for detecting injury risk.

*:W: week;GPS: Global Positioning System;sRPE: session rating of perceived exertion ;EWMA: Exponentially Weighted Moving Average;RA: Rolling Average;RPE: rating of perceived exertion;ACWR: Acute:Chronic Workload Ratio ;r: Correlation Coefficient

Table S10：Quality Assessment of the Included Studies

Author (Year)	S1: Representativeness of the Cohort	S2: Exposure Measurement	S3: Outcome Not Present at	S4: Clearly Defined Outcome	C1: Control for Confounding Factors (2	O1: Outcome Assessment Method	O2: Adequate Follow-up Duration	O3: Completeness of Follow-up	Total Score (/9)	Quality Rating

			Baseline		points)					
Bowen (2017)	1	1	1	1	0	1	1	1	7	Moderate
Bowen (2020)	1	1	0	1	0	1	1	1	6	Moderate
Arazi (2020)	0	1	0	1	0	1	0	1	4	Low
Carey (2017)	1	1	0	1	0	1	1	1	6	Moderate
Colby (2017)	1	1	0	1	2	1	1	1	8	High
Cross (2015)	1	1	0	1	2	1	1	1	8	High
Cummins (2019)	1	1	0	1	0	1	1	1	6	Moderate
Damji (2021)	0	1	0	1	2	0	1	1	6	Low
Delecroix (2018)	1	1	0	1	0	1	1	1	6	Moderate
Esmaeili (2018)	1	1	0	1	2	1	1	1	8	High
Fanchini (2018)	1	1	0	1	0	1	1	0	5	Moderate
Fousekis (2023)	1	1	0	1	2	1	1	0	7	Moderate
Hulin (2014)	1	1	0	1	0	1	1	0	5	Moderate
Hulin (2016a)	1	1	0	1	0	1	1	0	5	Moderate
Hulin (2016b)	1	1	0	1	0	1	1	0	5	Moderate
Iwasaki (2024)	1	1	0	1	2	1	1	0	7	Moderate
Jaspers (2017)	1	1	0	1	0	1	1	1	6	Moderate
Malone (2016a)	1	1	0	1	2	1	1	0	7	Moderate
Malone (2016b)	1	1	0	1	2	1	1	0	7	Moderate
Malone (2016)	1	1	0	1	0	1	1	0	5	Moderate
Malone (2018)	1	1	0	1	2	1	1	0	7	Moderate
McCall (2017)	1	1	0	1	0	1	1	1	6	Moderate
Mohr (2023)	1	1	1	0	0	1	1	0	5	Moderate
Murray (2016a)	1	1	1	0	0	1	1	0	5	Moderate
Murray (2016b)	1	1	1	0	0	1	1	0	5	Moderate

Nobari (2021)	1	1	0	1	0	0	1	0	4	Low
Nobari (2022)	1	1	0	1	0	1	1	0	5	Moderate
Oliveira (2021)	1	1	0	1	0	1	1	0	6	Moderate
Pajuelo (2021)	1	1	0	1	0	1	1	1	6	Moderate
Sampson(2018)	1	1	0	1	0	1	1	1	6	Moderate
Sampson (2019)	1	1	0	1	0	1	1	1	6	Moderate
Schumann (2023)	1	1	0	1	0	1	1	0	5	Moderate
Seco-Serna (2024)	1	1	0	1	0	0	1	1	5	Moderate
Stares (2017)	1	1	0	1	2	1	1	0	7	Moderate
Tiernan (2022)	1	1	1	1	0	1	1	1	7	Moderate
Timoteo (2018)	1	1	1	1	0	1	1	1	7	Moderate
Veiga (2021)	1	1	1	1	0	1	1	1	7	Moderate
Weiss (2017)	1	1	1	0	0	0	1	0	4	Low
West (2021)	1	1	1	0	2	1	1	1	8	High
Windt (2016)	1	1	1	0	2	1	1	0	7	Moderate
Wu (2024)	1	1	1	0	0	1	1	0	5	Moderate

* Overall quality ratings are reported as assigned in the final column.
